# DEC1 promotes breast cancer bone metastasis through transcriptional activation of CXCR4

**DOI:** 10.7555/JBR.39.20250031

**Published:** 2025-05-27

**Authors:** Ying Huo, Kaiao Chen, Zhiyi Qiang, Lan Lin, Wei Liu, Jian Yang

**Affiliations:** Department of Pharmacology, Nanjing Medical University, Nanjing, Jiangsu 210066, China

**Keywords:** breast cancer bone metastasis, differentiated embryonic chondrocyte expressed gene 1, CXCR4

## Abstract

Bone metastasis is the primary cause of mortality in breast cancer (BC) patients. The present study elucidates the functional role of the differentiated embryonic chondrocyte-expressed gene 1 (DEC1) in promoting BC-related bone metastasis. Analysis of patient-derived samples and public databases revealed a significant upregulation of DEC1 and CXCR4 in breast tumors compared with adjacent normal tissues, with elevated levels correlating with increased metastatic potential, suggesting their synergistic involvement in BC progression. Intracardiac injection experiments demonstrated that *Dec1*-WT 4T1 cells induced more severe osteolysis and larger metastatic lesions than *Dec1*-KD 4T1 cells. In MDA-MB-231 cells, *DEC1* overexpression (OE) upregulated CXCR4 and proliferation/migration-related genes, whereas *DEC1* knockdown reversed these effects. Notably, AMD3100, a specific CXCR4 antagonist, partially reversed the *DEC1*-OE-induced upregulation of CXCR4 and associated pro-metastatic genes. Mechanistically, DEC1 bound to the CXCR4 promoter region (−230 to −326) and activated its transcription, corroborated by ChIP-seq data. Furthermore, pharmacological inhibition of AKT (LY294002) or JAK2 (AZD1480), but not ERK (PD98059), attenuated DEC1-mediated CXCR4 upregulation, although all three inhibitors mitigated DEC1-driven migration-related gene expression. Additionally, DEC1 enhanced CXCL12 secretion from mesenchymal stromal cells and osteoblasts, amplifying the CXCR4/CXCL12 axis within the bone microenvironment. Collectively, our findings demonstrate that DEC1 promotes BC bone metastasis by directly transactivating CXCR4 expression, providing a molecular basis for targeting DEC1 to prevent and treat BC bone metastasis.

## Introduction

Breast cancer (BC) often occurs in women aged 40–60 ^[ 1] ^. BC is a prevalent malignancy among women, with an annual incidence of approximately two million new cases worldwide ^[ 2] ^. BC is generally not life-threatening in its early stages, but bone metastasis significantly contributes to mortality in BC patients ^[ 3] ^. It has been reported that approximately 70% of patients who died from BC have bone metastases ^[ 4– 5] ^. Bone metastasis leads to a succession of skeletal-related events, including pathological fractures such as spinal cord compression, severe pain in bones, hypercalcemia, and other skeletal complications that may cause patients to lose the ability to care for themselves, decrease their quality of life, and increase treatment costs ^[ 6] ^. In metastatic dissemination, BC cells must initially undergo epithelial-to-mesenchymal transition (EMT) to break down the surrounding tissue, subsequently enter the circulation *via* microvessels, and finally colonize the bone tissue ^[ 7– 8] ^. Bone metastasis occurs through the interaction of BC cells with the bone microenvironment and extensive bone resorption, which promotes the development of a pre-metastatic niche ^[ 9] ^.

Chemokines and their receptors promote communication between the disseminated tumor cells and the microenvironment of distant metastatic organs, facilitating organ-specific migration, seeding, and colonization (homing) ^[ 10] ^. During the homing process, tumor cells with increased CXCR4 expression exhibit a greater likelihood of surviving the circulation phase and are attracted to bone through CXCL12 secreted from the bone marrow stromal cells ^[ 11– 12] ^. Therefore, the CXCL12/CXCR4 axis may play a crucial role in the BC bone metastasis ^[ 11] ^. Several major signaling pathways, such as OPG/RANK/RANKL ^[ 13] ^, TGF-β ^[ 14] ^, PI3K-AKT-mTOR ^[ 15] ^, Wnt, and Hippo ^[ 16– 17] ^, are involved in BC bone metastasis. Transcription factors, including RunX2, NKX2-8, and FOXF2, play a critical role in the development of BC bone metastasis ^[ 8, 18– 19] ^. Elucidating the molecular mechanisms underlying BC osteotropism may aid in formulating predictive and therapeutic strategies for bone metastasis.

The transcription factor differentiated embryonic chondrocyte-expressed gene 1 (DEC1) is highly expressed in oral, thyroid, breast, gastric, and pancreatic cancers ^[ 20– 25] ^. Previous studies have demonstrated that DEC1 promotes EMT ^[ 26] ^, correlates with the malignancy and invasiveness of BC ^[ 22] ^, and is associated with bone metabolism ^[ 27– 29] ^. However, the role of DEC1 in BC bone metastasis remains unclear.

The current study employed *Dec1* ^+/+^ and *Dec1* ^−/−^ mice alongside gain- and loss-of-function experiments in MDA-MB-231 cells to define the mechanistic role of DEC1 and its molecular partners in breast cancer bone metastasis.

## Materials and methods

### Chemicals and reagents

Tartrate-resistant acid phosphatase (TRAP) Stain Kit (Cat. #D023-1-1) was from Nanjing Jiancheng Bioengineering Institute (Nanjing, China). CXCL12 enzyme-linked immunosorbent assay (ELISA) Kit (Cat. #KE10046) was from Proteintech (Wuhan, China). Color Development Kit (Cat. #I001-1-1) was from Nanjing Jiancheng (Nanjing, China). Dulbecco's modified Eagle's medium (Cat. #12800-017) and fetal bovine serum (FBS; Cat. #10270-106) were from Gibco. Transfection reagent GenJet (Cat. #SL100489) was from SignaGen (Maryland, USA). Enhanced chemiluminescence detection kit (Cat. #E422-02-AB) was from Vazyme (Nanjing, China). Flag-DEC1 and Flag-CMV2 plasmids were generously provided by Dr. Yan (University of Rhode Island, USA). The lentiviral *DEC1*-shRNA (three viral strains) and the corresponding vector (shRNA-con) were obtained from Genechem (Shanghai, China). Dual Luciferase Reporter Gene Assay Kit (Cat. #11402ES60) was purchased from YESEN (Shanghai, China). ADM3100 (Cat. #A2025) and actinomycin D (Act D, Cat. #A4448) were from apExBIO (Houston, USA). AZD1480 (Cat. #S2162), LY294002 (Cat. #S1105), and PD98059 (Cat. #S1177) were from Selleck (Houston, USA). Antibodies targeting CXCR4 (Cat. #BS2771, RRID: AB_1662477), MMP-9 (Cat. #BS67753, RRID: AB_3094871), MMP-1 (Cat. #BS1229, RRID: AB_1663982), VEGF (Cat. #BS6496, RRID: AB_2814865), E-cadherin (Cat. #BS79669, RRID: AB_3094872), vimentin (Cat. #P08670, RRID: AB_3094875), p-AKT (Cat. #BS4007, RRID: AB_1662951), AKT (Cat. #MB66248, RRID: AB_3094878), p-ERK1/2 (Cat. #AP0485, RRID: AB_2800355), ERK1/2 (Cat. #BS5517, RRID: AB_1661925), p-MEK1/2 (Cat. #4733, RRID: AB_1663182), and MEK1/2 (Cat. #BS3599, RRID: AB_1662317), as well as goat anti-rabbit IgG (H+L) fluorescein-5-isothiocyanate (FITC) (Cat. #BS10950, RRID: AB_3094880) and goat anti-mouse IgG (H+L) rhodamine (TRITC) (Cat. #BS11502, RRID: AB_3094881) were from Bioworld Technology (Nanjing, China). Anti-CXCL12 (Cat. #C121, RRID: B_2878404) was from Novoprotein (Suzhou, China). Anti-DEC1 (Cat. #sc-101023, RRID: AB_2065356), anti-SNAI1 (Cat. #sc-271977, RRID: AB_10709902), and anti-RAS (Cat. #sc-31, RRID: AB_628041) were from Santa Cruz (TX, USA). Anti-p-JAK2 (Cat. #YP0306, RRID: AB_3094867) was from Immunoway (TX, USA). Anti-JAK2 (Cat. #CY5287, RRID: AB_3094868) was from Abways (Beijing, China). Anti-CXCL12 (Cat. #17402-1-AP, RRID: AB_2878404), anti-N-cadherin (Cat. #22018-1-AP, RRID: AB_2813891), anti-Ki67 (Cat. #28074-1-AP, RRID: AB_2918145), anti-rabbit IgG (Cat. #30000-0-AP, RRID: AB_2819035), anti-mouse IgG (Cat. #SA00001-1, RRID: AB_2722565), and anti-β-actin (Cat. #81115-1-RR, RRID: AB_2923704) were from Proteintech.

### Tissue microarray

The human BC tissue microarray was obtained from AiFang Biological, comprising a total of 80 BC tissue samples and their paired adjacent normal tissues. DEC1 protein expression was assessed by immunohistochemistry in 80 BC samples and their paired adjacent normal tissues. Within the BC cohort, DEC1 expression levels were compared between smaller tumors (diameter < 3 cm; *n* = 34) and larger tumors (diameter ≥ 3 cm; *n* = 33). Additionally, DEC1 expression levels were compared between non-metastatic ( *n* = 19) and metastatic ( *n* = 30) BC cases.

### Participants

Eleven BC tissues from female BC patients with an average age of 52.9 years (ranging from 45 to 62 years) were collected from Sir Run Hospital. Histopathological examination was conducted following BC surgeries. The inclusion criteria were: (1) subjects who were diagnosed and confirmed *via* various methods, such as ultrasonography, mammography, and pathological examination; (2) subjects who underwent simple BC surgery only; and (3) subjects who signed the informed consent. The exclusion criteria were: (1) subjects with other malignancies or multiple primary tumors; (2) subjects who underwent BC surgery concurrently with other operations; and (3) subjects with uncertain pathological diagnosis. The study was conducted in accordance with the Declaration of Helsinki guidelines and approved by the Institutional Review Board of Nanjing Medical University (Approval No. 2020-602, Nanjing, China).

### Double-label immunofluorescence staining

For double-label immunofluorescence analysis, breast tissue sections were first treated with a 3% hydrogen peroxide solution and incubated in the dark for 15 min to block endogenous peroxidase activity, followed by antigen retrieval by heating in citrate buffer (pH 6.0) for 20 min. Then, the sections were blocked with 5% normal goat serum in PBS at room temperature for 1 h to reduce non-specific binding. Subsequently, the sections were treated with primary mouse anti-DEC1 (1∶200) and rabbit anti-CXCR4 (1∶200). After being washed with PBS three times, the sections were incubated with corresponding secondary antibodies, *i.e.*, goat anti-mouse TRITC (1∶500) and goat anti-rabbit FITC (1∶500) for 1 h. After being washed with PBS three times, the sections were mounted on coverslips and then examined using a fluorescent microscope (Olympus, Japan) with DP2-BSW acquisition software.

### Cell culture and treatment

Cell lines MCF10A (RRID: CVCL_0598), MDA-MB-231 (RRID: CVCL_0062), MCF-7 (RRID: CVCL_0031), SUM1315 (RRID: CVCL_5589), 4T1 (RRID: CVCL_0125), HUVEC (RRID: CVCL_2959), and MC3T3-E1 (RRID: CVCL_0409) were all obtained from the American Type Culture Collection (Manassas, VA, USA). Cells were incubated in DMEM with 5% FBS, 100 U/mL streptomycin, and 100 U/mL penicillin in a humidified incubator with 5% CO _2_ at 37 ℃, and the media were changed every two days.

For DEC1 overexpression, cells (1 × 10 ^6^/well) were plated in 6-well plates and cultured overnight, followed by transfection with either 1 μg of Flag- *DEC1* plasmid or Flag-CMV2 vector using Transfection Reagent Ⅱ. After being incubated for 5–6 h, the culture medium was replaced with fresh DMEM. The transfected cells were divided into three groups: Flag-CMV2, Flag- *DEC1*, and Flag- *DEC1* + ADM3100. The Flag- *DEC1* + ADM3100 group was treated with 40 μmol/L ADM3100, and the other two groups were treated with DMEM for 1 day.

For *DEC1* knockdown (KD), cells (1 × 10 ^5^) were infected with either the most effective of the three lentiviruses (LV-sh *DEC1*) or the control lentivirus (LV-Con). The infected cells were subjected to continuous incubation with 5 μg/mL puromycin in the medium for 15 days to obtain stable *DEC1*-knockdown cells (polyclones). The knockdown of DEC1 in 4T1 cells was validated by Western blotting ( ***Supplementary Fig. 1A***, available online).

### Animals

C57BL/6 mice (RRID: IMSR_JAX: 000664) were obtained from the Animal Core Facility of Nanjing Medical University (Nanjing, China). *Dec1* ^+/−^ mice (C57BL/6J background) were obtained from RIKEN BioResource Center (Japan). Heterozygous male mice ( *Dec1* ^+/−^) were bred with heterozygous female ones to generate wild type ( *Dec1* ^+/+^, WT) and *Dec1*-knockout ( *Dec1* ^−/−^, *Dec1-*KO) mice. Genotyping was conducted immediately after birth and verified again prior to the experiment ( ***Supplementary Fig. 1B***, available online). The female *Dec1-*KO and WT mice from the same littermate were selected for establishing BC bone metastasis. Mice were housed in a pathogen-free environment at the Animal Core of Nanjing Medical University (Nanjing, China) and maintained at a temperature of 20–26 ℃ and a humidity level of 30%–70%. They were kept under a standard 12∶12-h light-dark cycle and provided with standard rodent chow and water ad libitum. All animal experiments conducted in the present study were approved by the Institutional Animal Care and Use Committee (Approval No. IACUC-2103038).

### Construction of BC bone metastasis with intracardiac injection

BC bone metastasis with intracardiac injections was performed as previously described ^[ 30] ^. Specifically, murine BC 4T1 cells ( *Dec1*-WT or *Dec1*-KD, 1 × 10 ^6^) were injected into the left ventricle of female mice at the age of four weeks ( ***Supplementary Fig. 1C***, available online). Additionally, intracardiac injections of 4T1 cells (1 × 10 ^6^) were performed in WT or *Dec1*-KO female mice at the same age ( ***Supplementary Fig. 1D***, available online). After two months, all mice were anesthetized using chloral hydrate and then euthanized. The development of BC bone metastasis was monitored by X-ray (Faxitron MX-20). The hindlimb bones of mice were prepared for subsequent experiments.

### Bone histomorphometry analysis and immunohistochemical staining (IHC)

After being fixed with 4% paraformaldehyde (PFA) for 24 h and decalcified with 10% ethylene diamine tetraacetic acid for two to three weeks, the femurs of BC bone metastasis model mice were dehydrated with ethanol and xylene. The bone specimens were embedded in paraffin and sectioned at 5 µm using a paraffin microtome (Leica Microsystems, Wetzlar, Germany).

For TRAP staining, 5 μm-thick sections were prepared from paraffin-embedded mouse bone tissue. Following deparaffinization, TRAP activity was evaluated using a commercial TRAP staining kit (Jiancheng Bioengineering) according to the manufacturer's instructions.

For IHC staining, sections underwent dewaxing in xylene followed by rehydration in gradient ethanol. Subsequently, the rehydrated sections were treated with 3% H _2_O _2_ to block endogenous peroxidase activity at room temperature for 15 min. Then, after rinsing with PBS twice, the sections were treated with an antigen retrieval solution at 95 ℃ for 20 min. A 5% bovine serum albumin (BSA) solution was added to block the sections for 1 h. After that, the sections were incubated with primary antibodies against CXCR4 (1∶200), Ki67 (1∶200), VEGF (1∶200), MMP-9 (1∶200), or MMP-1 (1∶200) at 4 ℃ overnight. The sections were rinsed three times with PBS and then incubated with HRP-conjugated secondary antibodies at room temperature for 1 h. The enzyme-bound peroxidase was visualized using a DAB horseradish peroxidase color development kit. Immunoreactivity was observed under a light microscope (BX53, Olympus, Tokyo, Japan). The negative control was processed in parallel but without primary antibodies. Semi-quantitative analysis was performed using ImageJ 1.50i (NIH, Baltimore, MD, USA) by calculating the percentage of positively stained area in five randomly selected high-power fields per section. Each sample was analyzed using three non-consecutive sections, and each group included at least three mice.

### Cellular immunofluorescence staining

For CXCR4 staining, cells were fixed with 4% PFA, permeabilized with 0.2% Triton X-100, and blocked with 5% BSA. Thereafter, cells were incubated with a primary antibody (1∶200) against CXCR4 overnight, followed by FITC-conjugated secondary antibody for 1 h. Nuclei were counterstained with DAPI for 15 min, and images were captured using a confocal microscope (LSM710, Carl Zeiss Co., Germany).

In F-actin staining, cells were fixed with 4% PFA for 10 min at room temperature and then washed with PBS. Cells were permeabilized with 0.1% Triton X-100 for 5 min, followed by blocking with 1% BSA for 30 min. Cells were then stained with fluorescent phalloidin (100 nmol/L) for 30 min to visualize the cytoskeleton, and nuclei were labeled with DAPI. Images were captured using a confocal microscope (LSM710).

### Western blotting

Cell lysates (30, 20, 10, or 4 μg) as well as hindlimb lysates from model mice were separated by 7.5% SDS-polyacrylamide gel electrophoresis. The separated proteins were then transferred onto a polyvinylidene fluoride membrane. After being blocked with 5% non-fat milk, the membrane was incubated overnight with the following primary antibodies: anti-DEC1 (1∶500), anti-CXCR4 (1∶1000), anti-MMP-9 (1∶1000), anti-MMP-1 (1∶1000), anti-VEGF (1∶1000), anti-SANI1 (1∶500), anti-N-cadherin (1∶1000), anti-E-cadherin (1∶1000), anti-vimentin (1∶1000), anti-p-AKT (1∶1000), anti-AKT (1∶1000), anti-p-ERK1/2 (1∶1000), anti-ERK1/2 (1∶1000), anti-p-JAK2 (1∶1000), anti-JAK2 (1∶1000), anti-p-MEK1/2 (1∶1000), anti-MEK1/2 (1∶1000), anti-RAS (1∶1000), or anti-β-actin (1∶1000). After being washed with TBST three times, the membrane was incubated with the corresponding secondary antibody at a dilution of 1∶10000. The protein bands were detected using an enhanced chemiluminescence (ECL) system. Protein levels were quantified by densitometry analysis using ImageJ 1.50i (NIH, Baltimore, MD, USA, RRID: SCR_003070), normalized to β-actin, and expressed as the ratio of target protein to β-actin.

### Quantitative reverse transcription-PCR (RT-qPCR) analysis

Total RNA was extracted from cultured cells or hindlimb bone tissue of mice using TRIzol (Cat. #15596026CN; Invitrogen, USA). cDNA was synthesized by reverse transcription using the HiScript Ⅱ qRTSuperMix kit (Cat. #R222; Vazyme). Quantitative PCR was performed on the ABI 7300 Real-Time PCR System (Applied Biosystems, CA, USA) using a PCR kit (Cat. #Q331; Vazyme). ***Table 1*** presents the primer sequences for each reaction. The expression level of each gene was determined using the comparative Ct method and normalized to *GAPDH* (human) or *Gapdh* (mouse).

**Table 1:** Primer for qRT-PCR

Genes	Forward	Reverse
Homo- *GAPDH*	GGACCTGACCTGCCGTCTAG	GTAGCCCAGGATGCCCTTGA
Homo- *CXCR4*	GCAGCAGGTAGCAAAGTGAC	TGAAGTGTATATACTGATCCC
Homo- *DEC1*	ATCCAGCGGACTTTCGCTC	TAATTGCGCCGATCCTTTCTC
Mus- *Gapdh*	GGATGCTGCCCTTACCC	GTTCACACCGACCTTCACC
Mus- *Cxcr4*	AGGAAACTGCTGGCTGAA	GGTAAAGGCGGTCACAGA
Mus- *Dec1*	GCCAAGGGATCAGAAGG	CAATTCACCTCCATAGCCAC
Mus- *Cxcl12*	GACACACAAAGCCCAAAGA	AGGAAGGTAAAACCCCACA

### Colony formation assay

To investigate the role of DEC1 and CXCR4 in BC cell proliferation, MDA-MB-231 cells (1500/well) from the Flag-CMV2, Flag- *DEC1*, and Flag- *DEC1* + AMD3100, LV-shCon, and LV-sh *DEC1* groups were plated in 6-well plates overnight, respectively. The Flag- *DEC1* + AMD3100 group was treated with 40 μmol/L AMD3100, while the other groups received the same amount of PBS. Cells were incubated at 37 ℃ with 5% CO _2_ and 95% humidity for 10 days, with medium changed on day 5. After that, cells were fixed in 4% PFA for 30 min and then stained with crystal violet to visualize the colony formation. Colonies were counted in five randomly selected high-power fields per well using ImageJ 1.50i. All data were obtained from at least three independent experiments.

### Wound-healing assay

To investigate the role of DEC1 and CXCR4 in BC cell migration, MDA-MB-231 cells (1 × 10 ^6^/well) from the Flag-CMV2, Flag- *DEC1*, Flag- *DEC1* + AMD3100, LV-shCon, and LV-sh *DEC1* groups were plated in 6-well plates overnight. Wounds were created using a 10-μL pipette tip and were gently washed with PBS to remove the scraped cells. Cells were cultured in a serum-free medium, and images were captured. Then, the Flag- *DEC1* + AMD3100 group was treated with AMD3100 (40 μmol/L), while the other groups received the same amount of PBS. Cells were cultured in the serum-free medium for 24 h, after which wound closure was imaged using an inverted system microscope (Olympus, Japan). The wound area was measured using ImageJ 1.50i. Wound closure percentage was calculated as: [(wound area at 0 h − wound area at 24 h) / wound area at 0 h] × 100%. All data were derived from at least three independent experiments.

### Transwell migration assay

To investigate the effect of DEC1 and CXCR4 on migration in response to CXCL12 in BC cells, the transfected or infected MDA-MB-231 cells (1 × 10 ^5^) from the Flag-CMV2, Flag- *DEC1*, Flag- *DEC1* + AMD3100, LV-shCon, and LV-sh *DEC1* groups were plated in the top chamber of a Transwell insert (pore size 8.0 μm) in DMEM medium, and the bottom chamber (without matrix gel) was supplemented with 2.5% FBS DMEM containing 100 ng/mL CXCL12. Cells were incubated at 37 ℃ with 5% CO _2_ and 95% humidity for 24 h. Migrated cells in the bottom chamber were fixed in 4% PFA, stained with crystal violet, and quantified using ImageJ 1.50i. Data were derived from at least three independent experiments.

To investigate the role of DEC1 in osteoblasts as a tumor microenvironment (MC3T3-E1) in regulating BC cell migration, we first obtained conditioned medium from MC3T3-E1 cells with DEC1 overexpression or knockdown, which had been cultured for 24 h. Then, 1 × 10 ^5^ MDA-MB-231 cells were plated in the top chamber of a Transwell insert with no matrix gel in the bottom chamber. The cells were cultured with the aforementioned conditioned medium in an incubator with 95% humidity, 37 ℃, and 5% CO _2_ for 24 h. Next, the cells that migrated from the top chamber to the bottom chamber were fixed with 4% PFA and stained with crystal violet. The migrated cells were quantified using ImageJ 1.50i. Meanwhile, we measured the conditioned media for CXCL12 content using an ELISA kit. All data were derived from at least three independent experiments.

### Angiogenesis

To investigate the role of DEC1 and CXCR4 in BC cells in enhancing angiogenesis, we conducted angiogenesis assays using different tumor-conditioned media (TCM). First, cells from the Flag-CMV2, Flag- *DEC1*, Flag- *DEC1* + AMD3100, LV-shCon, and LV-sh *DEC1* groups were plated in 6-well plates and incubated overnight. Then, the medium was refreshed with serum-free DMEM medium for 24 h. The medium was centrifuged and filtered to obtain the TCM. Cells were harvested and lysed to detect VEGF, MMP-1, and MMP-9 protein levels by Western blotting. For the tube formation assay, human umbilical vein endothelial cells (HUVECs, 2 × 10 ^4^) were plated in 96-well plates pre-coated with 50 μL Matrigel and incubated with 200 μL of TCM from each group for 6 h. The tube formation images were captured under a microscope (100×). The tubes were quantified using ImageJ 1.50i. All data were derived from at least three independent experiments.

### Chromatin immunoprecipitation sequencing (ChIP-seq)

The transfected MDA-MB-231 cells (Flag- *DEC1* or Flag-CMV2, 3×10 ^8^) were cross-linked with 1% fresh formaldehyde at room temperature for 10 min. The cross-linking reaction was then neutralized with glycine. Tsingke Biotechnology (Tsingke Biotechnology Co., Ltd., Beijing, China) performed the ChIP-seq experiments. They annotated the peak of each sample's call and the peak of the difference using ChIPseeker and analyzed the gene set enrichment using clusterProfiler ^[ 31– 33] ^.

### Plasmid construction

Flag- *DEC1* and a set of mutated Flag- *DEC1* plasmids were provided by Dr. Yan (University of Cincinnati, USA). Based on ChIP-seq data showing that DEC1 may bind to the *CXCR4* promoter, we constructed a full-length *CXCR4* promoter reporter and several deleted or mutant *CXCR4* promoter reporters. The human CXCR4 promoter reporter (−568/+1), which contains DEC1 binding sequences (SP1 sites), was constructed by cloning a 568-bp fragment upstream of the transcription start site into the pGL3-basic vector (Promega, Beijing, China; RRID: Addgene_11994). Plasmids with deletions and mutations were constructed by PCR with primers listed in ***Table 2***. The fragments containing these elements were amplified by PCR using primers that incorporated suitable restriction enzyme sites (NheⅠ/HindⅢ) to enable subsequent ligation. The sequences of all *CXCR4* promoter reporters were verified by DNA sequencing.

**Table 2:** Primers used for preparation of mutated constructs of CXCR4-luc reporter gene

Genes	Forward
*CXCR4* (568 bp)	CTA GCTAGCAGTGGCTGCATGTGTCTCC
*CXCR4* (415 bp)	CTA GCTAGCTGTCTTGGAGCGAGTTACATTGT
*CXCR4* (326 bp)	CTA GCTAGCACTTCGGGGTTAAGCGCCTGG
*CXCR4* (203 bp)	CTA GCTAGCGTAGGCAGAGGGCGGGA
*CXCR4* (109 bp)	CTA GCTAGCCCGCCGTCCGAAGCGCG
The underlined letters indicate the restriction sites.	

### Dual-luciferase reporter activity assay

MDA-MB-231 cells (8 × 10 ^4^/well) were seeded in 48-well plates. Transfection mixtures contained 50 ng of *CXCR4*-Luc, 10 ng of Flag- *DEC1* or LV-sh *DEC1*, and 5 ng of Null-Renilla reniformis luciferase plasmid. The vector plasmid was adjusted to an equal amount of DNA in each transfection. At 24 h post-transfection, cells were lysed using 1× passive lysis buffer (Promega, Madison, WI, USA) and subjected to two freeze-thaw cycles to ensure complete lysis. Promoter reporter activities were measured using a commercial assay system (Cat. #11402ES60, YEASEN, China). Normalized luciferase activity was calculated as the ratio of *CXCR4*-Luc activity to Null-Renilla luciferase activity. Relative luciferase activity was calculated as the ratio of normalized luciferase activity of Flag- *DEC1* or LV-sh *DEC1* to normalized luciferase activity of vector. All data were derived from at least three independent experiments.

### Statistical analysis

We analyzed the data using one- or two-way ANOVA, followed by Tukey's or Sidak's post hoc tests for multiple comparisons (two-tailed). We tested the paired comparison data using Student's *t*-test (IBM SPSS Statistics software, version 25.0, RRID: SCR_002865). Data were expressed as mean ± standard deviation. *P* < 0.05 was considered statistically significant.

## Results

### Upregulated DEC1 and CXCR4 expression in human BC tissues

**Figure 1:**
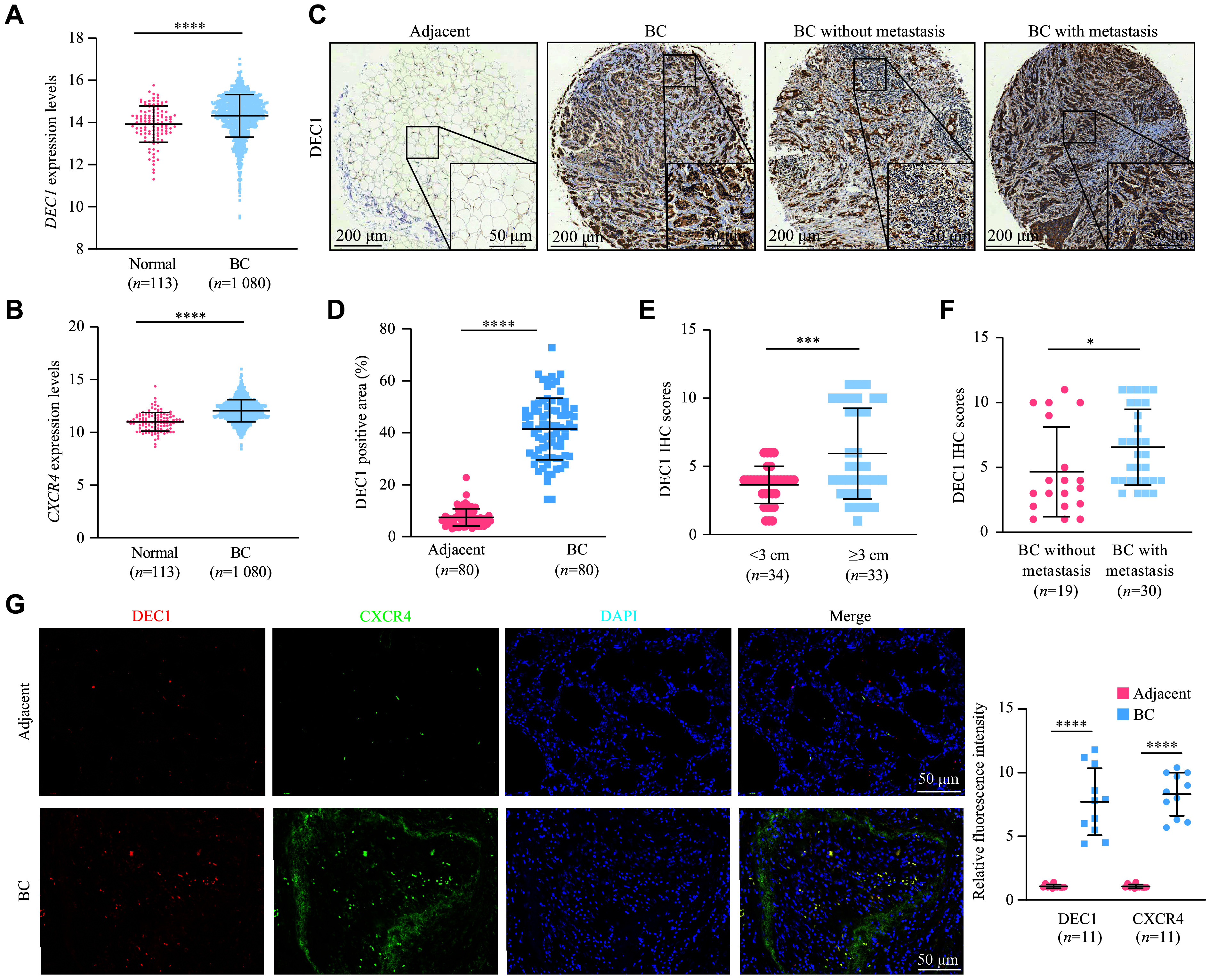
Upregulated DEC1 and CXCR4 expression in human BC tissues. A and B: The expression levels of *DEC1* and *CXCR4* in breast cancer (BC; *n* = 1080) and normal breast tissues ( *n* = 113) were analyzed using the TCGA database. C–F: DEC1 levels in tissue microarrays of BC samples ( *n* = 80) were examined by immunohistochemistry (IHC). Representative IHC staining of DEC1 in BC and adjacent normal tissues (C). Comparison of the DEC1 positive area in BC and adjacent normal tissues (D). Comparison of DEC1 IHC scores in smaller and larger BC specimens (E). Comparison of DEC1 IHC scores in BC specimens with or without distant metastasis (F). G: Double-label immunofluorescence staining of DEC1 and CXCR4 in BC tissues ( *n* = 11). Data are presented as mean ± standard deviation. ^*^ *P* < 0.05, ^***^ *P* < 0.001, and ^****^ *P* < 0.0001 by Student's *t*-test. Abbreviation: BC, breast cancer.

According to the analysis of the clinical BC samples through the human cancer database TCGA ( https://portal.gdc.cancer.gov/), *DEC1* mRNA levels were significantly upregulated in BC tissues ( *n* = 1080), compared with normal breast tissues ( *n* = 113; ***Fig. 1A***). In the same batch of samples, the *CXCR4* mRNA levels were also significantly upregulated in BC tissues ( *n* = 1080), compared with normal breast tissues ( *n* = 113; ***Fig. 1B***). Using tissue microarrays containing 80 matched primary/metastatic BC tissue and adjacent normal tissue pairs, we observed significantly stronger DEC1 immunostaining in BC tissues than in adjacent normal tissues ( ***Fig. 1C*** [left two panels] and ***Fig. 1D***). Notably, DEC1 protein levels were significantly elevated in larger tumor specimens (diameter ≥ 3 cm) compared with smaller tumors (diameter < 3 cm; ***Fig. 1E***). Additionally, DEC1 protein levels were significantly increased in BC with metastasis compared with those without metastasis ( ***Fig. 1C*** [right two panels] and ***Fig. 1F***). Consistent with the bioinformatics and tissue microarray results, dual-label immunofluorescence staining revealed a significant upregulation of both DEC1 and CXCR4 in BC tissues compared with adjacent normal tissues ( ***Fig. 1G***). Collectively, these data indicate that high DEC1 levels in BC tissues are accompanied by increased CXCR4 expression.

### *Dec1* knockdown in 4T1 cells protected against BC bone metastasis and reduced CXCR4 expression in mouse models

**Figure 2:**
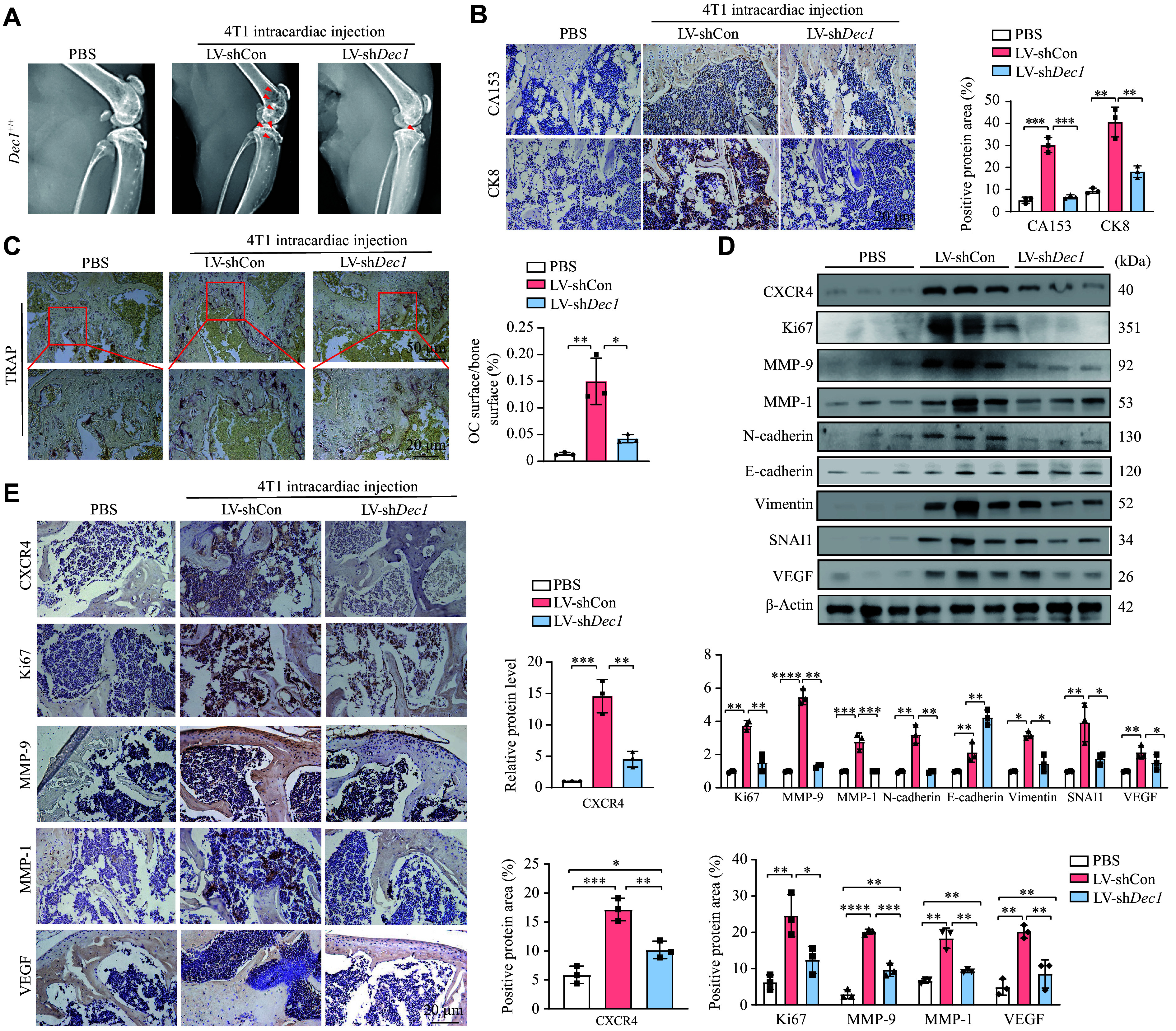
Knockdown of *Dec1* in 4T1 cells protected against breast cancer (BC) bone metastasis and reduced CXCR4 expression in mice. Four-week-old wild-type female mice received an intracardiac injection of 4T1 mouse BC cells (4T1- *Dec1*-WT [LV-shCon] or 4T1- *Dec1*-KD [LV-sh *Dec1*], 1 × 10 ^6^) for two months. The control group received an equal volume of PBS *via* the same method. *n* = 6 for each group. A: The bone destruction and osteolytic bone metastasis were detected by X-ray. B: The expression levels of CK8 (a glandular epithelium marker) and CA153 (a specific BC marker) in hindlimb bone tissues were detected by immunohistochemistry (IHC). C: The TRAP-positive cells and OC surface/bone surface (%) in hindlimb bone tissues were examined by IHC. D: Protein levels of CXCR4, Ki67, MMP-1, MMP-9, N-cadherin, E-cadherin, vimentin, SNAI1, and VEGF in hindlimb bone tissues were detected by Western blotting. E: Protein levels of CXCR4, Ki67, MMP-1, MMP-9, and VEGF in hindlimb bone tissues were detected by IHC. Data are presented as mean ± standard deviation ( *n* = 3 in each group). ^*^ *P* < 0.05, ^**^ *P* < 0.01, ^***^ *P* < 0.001, and ^****^ *P* < 0.0001. Scale bar = 20 μm. Data were analyzed using two-way ANOVA, and differences between groups were analyzed using Student's *t*-test.

To investigate the role and underlying mechanism of DEC1 in BC bone metastasis, we injected stable *Dec1*-WT or *Dec1*-KD 4T1 cells into the left ventricle of four-week-old female wild-type mice. An equal volume of PBS was injected using the same method as a negative control ( ***Supplementary Fig. 1C***). Compared with the negative control mice, those injected with *Dec1*-WT 4T1 cells intracardially exhibited severe bone destruction and osteolytic bone metastasis ( ***Fig. 2A***). Conversely, mice injected with *Dec1*-KD 4T1 cells intracardially displayed only minor bone destruction ( ***Fig. 2A***). IHC analysis revealed the protective effect of *Dec1* knockdown against BC bone metastasis, as indicated by the significantly reduced CK8 (a glandular epithelium marker) and CA153 (a specific BC marker) protein levels in the *Dec1*-KD group compared with the *Dec1*-WT group ( ***Fig. 2B***). Additionally, TRAP staining of the femur showed a significant increase in TRAP ^+^ cells in the *Dec1*-WT group, whereas only a slight increase was detected in the *Dec1*-KD group compared with the PBS group ( ***Fig. 2C***). Notably, the relative osteoclastic area (TRAP ^+^ cells) in the *Dec1*-KD group was significantly decreased compared with the *Dec1*-WT group ( ***Fig. 2C***). Although CXCR4 protein levels were increased in both model groups ( *Dec1*-WT and *Dec1*-KD) compared with the PBS group, they were significantly lower in the *Dec1*-KD group than in the *Dec1*-WT group. The changes were demonstrated by Western blotting and IHC analyses ( ***Fig. 2D*** [first line] and ***2E*** [first line]). Furthermore, MMP-9, MMP-1, and N-cadherin protein levels were significantly increased in the *Dec1*-WT group compared with the PBS group, while this increase was attenuated in the *Dec1*-KD group ( ***Fig. 2D***), with consistent results validated by IHC ( ***Fig. 2E***). Additionally, the ratios of p-AKT/AKT, p-JAK2/JAK2, and p-ERK1/2/ERK1/2 in the femur were significantly elevated in the *Dec1*-WT group compared with the PBS group, while these increases were partially or completely abolished in the *Dec1*-KD group ( ***Supplementary Fig. 2***, available online). Collectively, these results indicate that *Dec1* knockdown inhibits BC bone metastasis by downregulating CXCR4 expression, potentially mediated by AKT, JAK2, and ERK1/2 pathways.

### *In vitro* analysis revealed that DEC1 increased CXCR4 levels in BC cells

**Figure 3:**
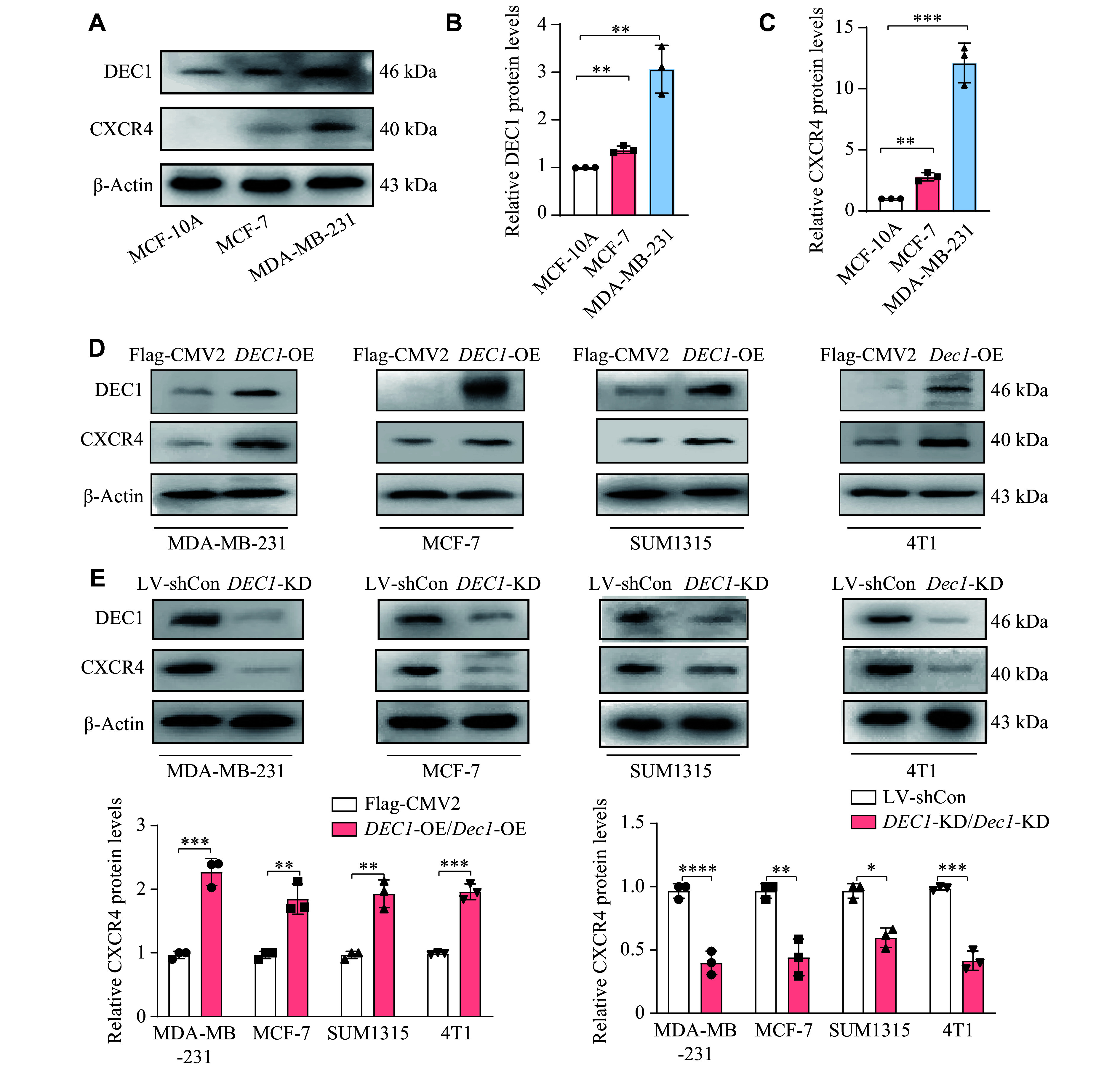
DEC1 increased the chemokine receptor CXCR4 in the BC cells. A–C: The DEC1 and CXCR4 expression levels in MCF-10A, MCF-7, and MDA-MB-231 cells. D: The CXCR4 expression levels were detected by Western blotting after overexpression of DEC1 in MDA-MB-231, MCF-7, SUM1315, and 4T1 cells. E: The CXCR4 expression levels were detected by Western blotting after knockdown of DEC1 in MDA-MB-231, MCF-7, SUM1315, and 4T1 cells. The data are presented as mean ± standard deviation (all experiments were repeated at least three times). ^*^ *P* < 0.05, ^**^ *P* < 0.01, ^***^ *P* < 0.001, and ^****^ *P* < 0.0001, comparisons are shown in the figure. Data were analyzed using one-way ANOVA, and differences between groups were analyzed using Student's *t*-test. Abbreviations: BC, breast cancer; KD, knockdown; OE, overexpression.

Given that both DEC1 and CXCR4 were significantly upregulated in human BC tissues, and *Dec1* knockdown suppressed BC bone metastasis and reduced CXCR4 expression in the mouse BC bone metastasis model, we further investigated the regulatory relationship between DEC1 and CXCR4 in BC cells. First, we examined DEC1 and CXCR4 expression in normal breast epithelial cells (MCF-10A) and two BC cell lines (MDA-MB-231 and MCF-7). Comparative analysis revealed a distinct expression pattern: MDA-MB-231 cells exhibited the highest levels of both DEC1 and CXCR4, followed by MCF-7 cells, while MCF-10A showed minimal expression ( ***Fig. 3A***– ***3C***). To further investigate this expression pattern, we performed *DEC1* knockdown and overexpression experiments in three human BC cell lines (MDA-MB-231, MCF-7, and SUM1315) and one murine BC cell line (4T1). As shown in ***Fig. 3D***, DEC1 overexpression significantly upregulated CXCR4 expression across all tested cell lines. Conversely, *DEC1* knockdown significantly downregulated CXCR4 expression ( ***Fig. 3E***). These findings indicate that DEC1 positively regulates CXCR4 expression in BC cells.

### CXCR4 involved in DEC1-induced BC cell proliferation and migration

**Figure 4:**
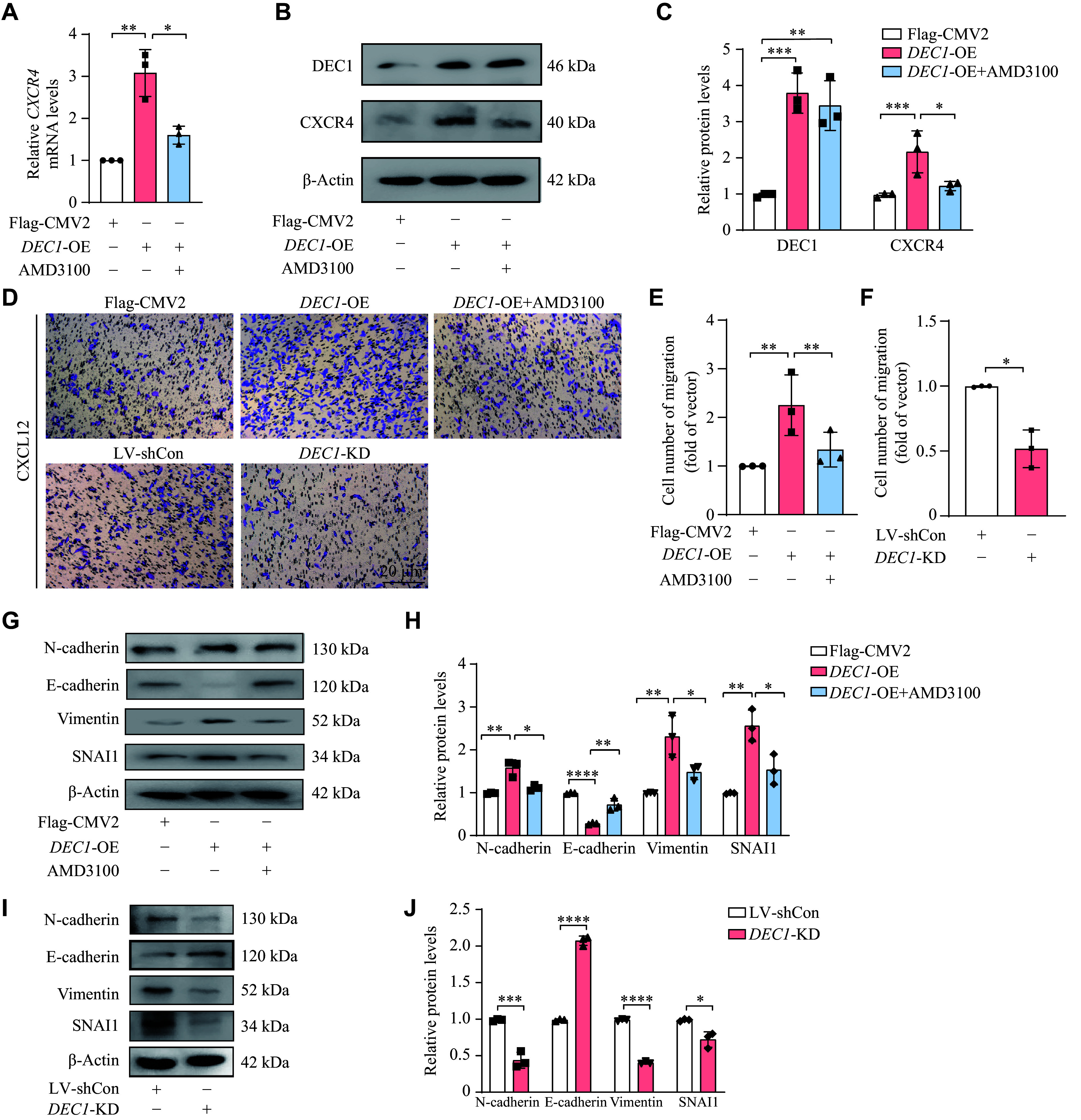
CXCR4 was involved in the increase of migration of MDA-MB-231 cells induced by *DEC1* overexpression. MDA-MB-231 cells were seeded into 6-well plates and cultured overnight. For *DEC1* overexpression (OE), the cells were transfected with either Flag-CMV2 or Flag- *DEC1*. The transfected cells were divided into three groups: Flag-CMV2, Flag- *DEC1*, and Flag- *DEC1* + AMD3100. Cells in the Flag- *DEC1* + AMD3100 group were treated with AMD3100, while the other two groups received DMSO for 24 h. For *DEC1* knockdown (KD), cells were infected with LV-shCon or LV-sh *DEC1* for 24 h. A–C: The effect of AMD3100 on the *DEC1*-OE-induced upregulation of CXCR4. D–F: The impact of *DEC1*-OE or KD on breast cancer (BC) cell migration. G–J: The impact of *DEC1*-OE or KD on the expression of migration-associated proteins, including N-cadherin, vimentin, SNAI1, and E-cadherin by Western blotting. The data are presented as mean ± standard deviation (all experiments were repeated at least three times). ^*^ *P* < 0.05, ^**^ *P* < 0.01, ^***^ *P* < 0.001, and ^****^ *P* < 0.0001, comparisons are shown in the figure. Data were analyzed using two-way ANOVA, and differences between groups were analyzed using Student's *t*-test.

To determine whether CXCR4 mediates DEC1-induced BC cell proliferation and migration, we treated *DEC1*-OE MDA-MB-231 cells with AMD3100, a specific CXCR4 antagonist. We found that *DEC1*-OE significantly increased CXCR4 expression, which was partially reversed by AMD3100 treatment ( ***Fig. 4A***– ***4C***). Subsequent analyses examined CXCR4 expression levels along with cell proliferative and migratory capacities. As shown in ***Supplementary Fig. 3A***– ***3I*** (available online), *DEC1*-OE significantly upregulated CXCR4 expression, accelerated wound closure, and enhanced colony formation; notably, AMD3100 treatment significantly abrogated these pro-tumorigenic effects. Conversely, *DEC1*-KD significantly downregulated CXCR4 expression and impaired both wound healing and colony formation. Consistent with these findings, *DEC1*-OE promoted cell migration in a CXCR4-dependent manner, as AMD3100 treatment effectively reversed this effect; however, *DEC1*-KD also suppressed cell migratory capacity ( ***Fig. 4D***– ***4F***, ***Supplementary Fig. 1A***). These observations were further validated by F-actin staining, which revealed corresponding changes in cytoskeletal organization ( ***Supplementary Fig. 3J***– ***3M***). To explore the molecular mechanisms underlying these phenotypic changes, we examined key regulatory proteins involved in BC cell proliferation and migration. *DEC1*-OE induced characteristic EMT markers, including upregulation of N-cadherin, vimentin, and SNAI1, with concurrent downregulation of E-cadherin ( ***Fig. 4G*** and ***4H***). Moreover, *DEC1*-OE significantly increased the levels of proliferation markers (Ki67 and PCNA) and stemness factors (NANOG and OCT4; ***Supplementary Fig. 3N*** and ***3O***). However, *DEC1*-KD showed the opposite effects on these protein levels ( ***Fig. 4I*** and ***4J***, ***Supplementary Fig. 3P*** and ***3Q***). Importantly, AMD3100 treatment effectively reversed these DEC1-mediated effects, normalizing the expression levels of all examined markers ( ***Fig. 4G*** and ***4H***, ***Supplementary Fig. 3N*** and ***3O***). Taken together, these results indicate that CXCR4 plays a crucial role in DEC1-induced BC cell proliferation and migration.

### CXCR4 involved in DEC1-mediated enhancement of angiogenesis

**Figure 5:**
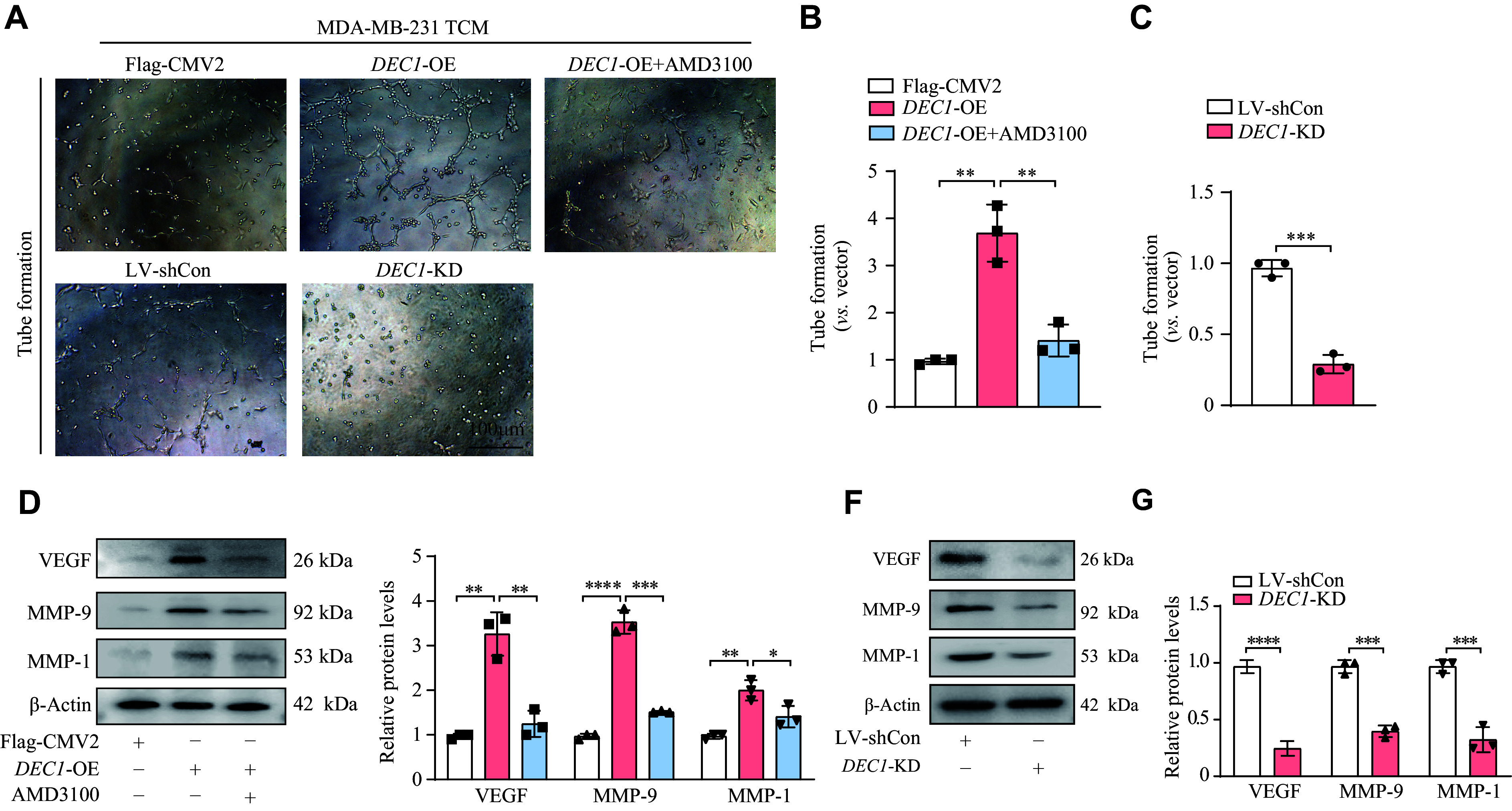
CXCR4 was involved in the promotion of angiogenesis in HUVECs mediated by DEC1. Tumor-conditioned media (TCM) were collected from the five experimental groups: Flag-CMV2, Flag- *DEC1*, Flag- *DEC1* + AMD3100, LV-shCon, and LV-sh *DEC1*. Human umbilical vein endothelial cells (HUVECs) (2 × 10 ^4^) were seeded into 96-well plates pre-coated with Matrigel and treated with 200 μL of the corresponding TCM from each group for 6 h. Tube formation was assessed microscopically, and images were captured. Subsequently, cells from these five groups were harvested to analyze the expression of VEGF, MMP1, and MMP9 by Western blotting. A–C: The impact of TCM from MDA-MB-231 cells with *DEC1* overexpression (OE) or knockdown (KD) on the angiogenesis of HUVECs. D–G: TCM from MDA-MB-231 cells with *DEC1*-OE or KD altered the expression levels of angiogenesis-related proteins, including VEGF, MMP1, and MMP9. Data are presented as mean ± standard deviation (all experiments were repeated at least three times). ^*^ *P* < 0.05, ^**^ *P* < 0.01, ^***^ *P* < 0.001, and ^****^ *P* < 0.0001, comparisons are shown in the figure. Data were analyzed using two-way ANOVA, and differences between groups were analyzed using Student's *t*-test.

To sustain their unlimited growth, BC cells from primary lesions must establish functional vascular networks that support the substantial energy demands of both proliferation and metastatic dissemination ^[ 34] ^. Given the critical role of angiogenesis in these processes, we investigated whether CXCR4 mediates DEC1-driven angiogenesis in BC. We performed HUVEC tube formation assays using TCM collected from MDA-MB-231 cells in the Flag-CMV2, Flag- *DEC1*, Flag- *DEC1* + AMD3100, LV-shCon, and LV-sh *DEC1* groups. Tube formation assays revealed that DEC1 significantly modulates tumor-induced angiogenesis. TCM from *DEC1*-OE cells significantly enhanced HUVEC tube formation ( ***Fig. 5A*** [top] and ***5B***), while TCM from *DEC1*-KD cells significantly inhibited angiogenic capacity ( ***Fig. 5A*** [bottom] and ***5C***). Notably, AMD3100 treatment partially or completely reversed the effects of TCM, suggesting that this process is dependent on CXCR4 ( ***Fig. 5A*** [top] and ***5B***). Consistent with these functional observations, *DEC1*-OE significantly upregulated key angiogenesis-related factors, including VEGF, MMP-9, and MMP-1 ( ***Fig. 5D*** and ***5E***), while *DEC1*-KD significantly downregulated their expression ( ***Fig. 5F*** and ***5G***). Notably, AMD3100 treatment significantly normalized the elevated levels of these pro-angiogenic proteins induced by *DEC1*-OE ( ***Fig. 5D*** and ***5E***). These data demonstrate that CXCR4 is involved in DEC1-mediated enhancement of angiogenesis.

### DEC1 acted as a novel transcriptional regulator of CXCR4 in BC

**Figure 6:**
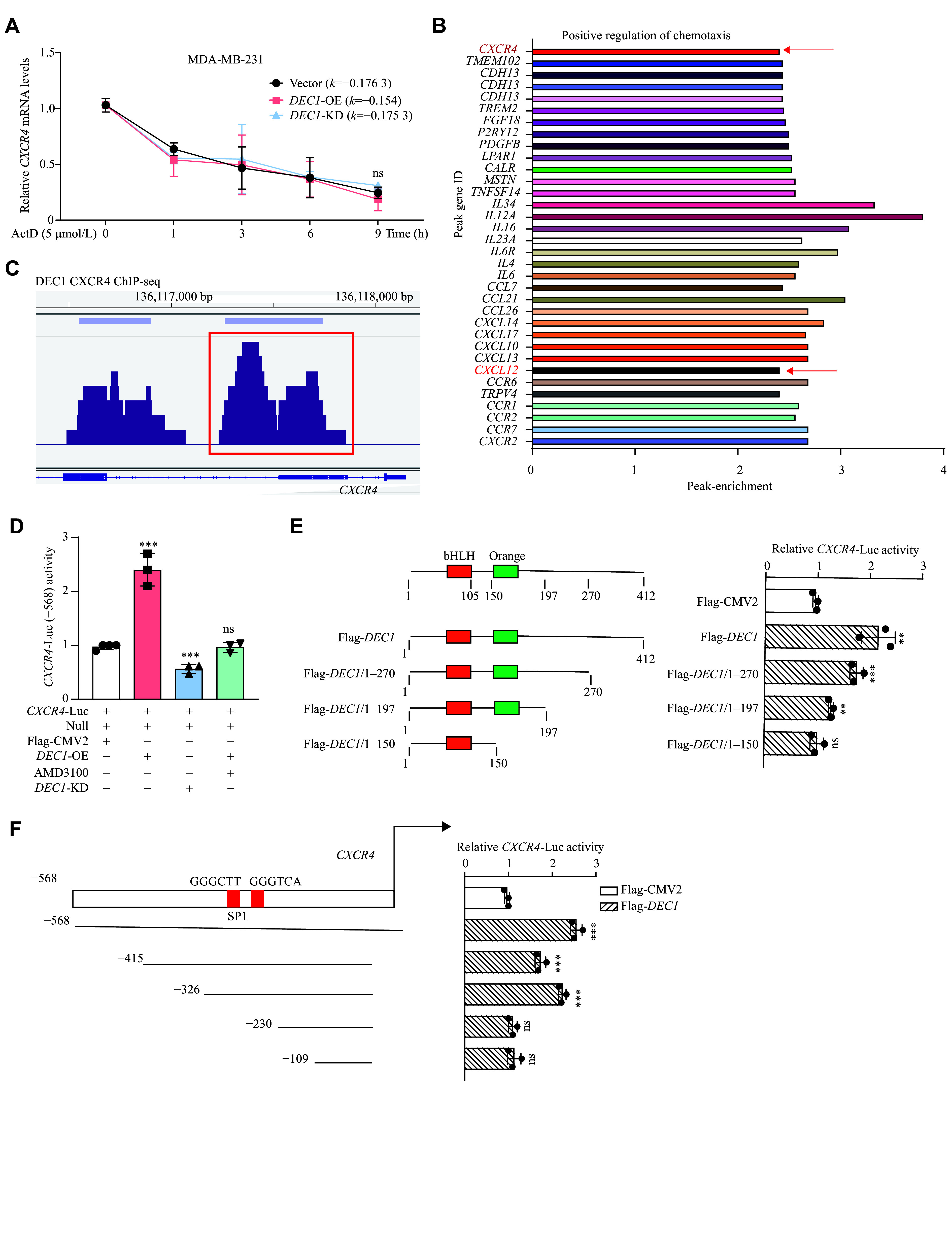
DEC1 transcriptionally promoted the CXCR4 expression in MDA-MB-231 cells. A: MDA-MB-231 cells were seeded into 6-well plates and cultured overnight. Cells were divided into three groups: Vector (infected with LV-shCon and transfected with Flag-CMV2), *DEC1*-OE (transfected with Flag-DEC1), and *DEC1*-KD (infected with LV-sh *DEC1*). Then, the latter two groups were treated with ActD (5 μmol/L), and the former group with an equal volume of phosphate-buffered saline for 0, 1, 3, and 6 h. CXCR4 mRNA stability after DEC1 modulation was measured by qPCR. B and C: MDA-MB-231 cells were seeded into 6-well plates and cultured overnight. Following a 24-h transfection with either Flag-CMV2 or Flag- *DEC1*, the cells were harvested for ChIP-seq analysis. The identified *DEC1* enrichment at genomic loci associated with chemokines by ChIP-seq analysis (B). Direct DEC1 binding to the *CXCR4* promoter region by ChIP-seq analysis (C). D: The effect of DEC1 on the *CXCR4*-Luc reporter activity. E: Differential effect of wild type (WT) and various mutated *DEC1* on *CXCR4* promoter. F: Differential repression of *DEC1* (WT) on the *CXCR4*-Luc and deleted mutants of *CXCR4*-Luc. Data are mean ± standard deviation (all experiments were repeated at least three times). ^**^ *P* < 0.01, ^***^ *P* < 0.001, and ^ns^ *P* > 0.05, comparisons are shown in the figure. Data were analyzed using one-way ANOVA, and differences between groups were analyzed using Student's *t*-test. Abbreviations: KD, knockdown; OE, overexpression.

Having established that DEC1 overexpression increases *CXCR4* mRNA levels ( ***Fig. 4A***), we next investigated whether this regulation occurred through transcriptional activation or mRNA stabilization. Actinomycin D chase assays revealed that no significant differences were observed in *CXCR4* mRNA decay rates between the Vector, *DEC1*-OE, and *DEC1*-KD groups of MDA-MB-231 cells ( ***Fig. 6A***), ruling out post-transcriptional regulation through mRNA stability. ChIP-seq analysis identified *DEC1* enrichment at genomic loci associated with chemotaxis regulators, particularly *CXCR4* and *CXCL12* ( ***Fig. 6B***). Detailed examination demonstrated direct binding of DEC1 to the CXCR4 promoter region (−568/+1; ***Fig. 6C***). Functional validation showed that *DEC1*-OE enhanced, while *DEC1*-KD reduced, the activity of the CXCR4 (−568/+1) promoter reporter ( ***Fig. 6D***). Notably, CXCR4 antagonist AMD3100 significantly abrogated DEC1-mediated promoter activation ( ***Fig. 6D***), indicating the functional dependence on CXCR4 signaling. The demonstrated activation of CXCR4 promoter activity by DEC1 provides conclusive evidence of its transcriptional regulatory function.

To identify critical DEC1 domains for this regulation, we generated a series of C-terminal deletion mutants ( ***Fig. 6E***, left) of DEC1 and assessed their ability to activate the CXCR4 (−568/+1) promoter. Notably, while full-length DEC1 (Flag- *DEC1*/1–412) and truncations Flag- *DEC1*/1–270 and Flag- *DEC1*/1–197 retained promoter-activating capacity, the Flag- *DEC1*/1–150 mutant (lacking the ligand-binding domain) completely lost promoter activity ( ***Fig. 6E***, right). These results precisely mapped the regulatory function to DEC1's ligand-binding domain (residues 150–197), establishing it as essential for CXCR4 transactivation.

To identify the CXCR4 promoter elements required for DEC1-mediated transactivation, we generated four progressive deletion constructs through systematic mutagenesis of the CXCR4 promoter region (−415 to +1; ***Fig. 6F***, left). Functional assays revealed that DEC1 significantly activated both the full-length (−415/+1) and −326/+1 truncated promoters, but failed to stimulate the −230/+1 and −109/+1 constructs ( ***Fig. 6F***, right). These results precisely localized the DEC1-responsive element to a 96-bp region between −326 bp and −230 bp upstream of the transcription start site.

### DEC1 upregulated CXCR4 expression and enhanced protein levels of key metastasis-associated markers in breast cancer cells, through activating PI3K/AKT, JAK/STAT, and MEK/ERK pathways

**Figure 7:**
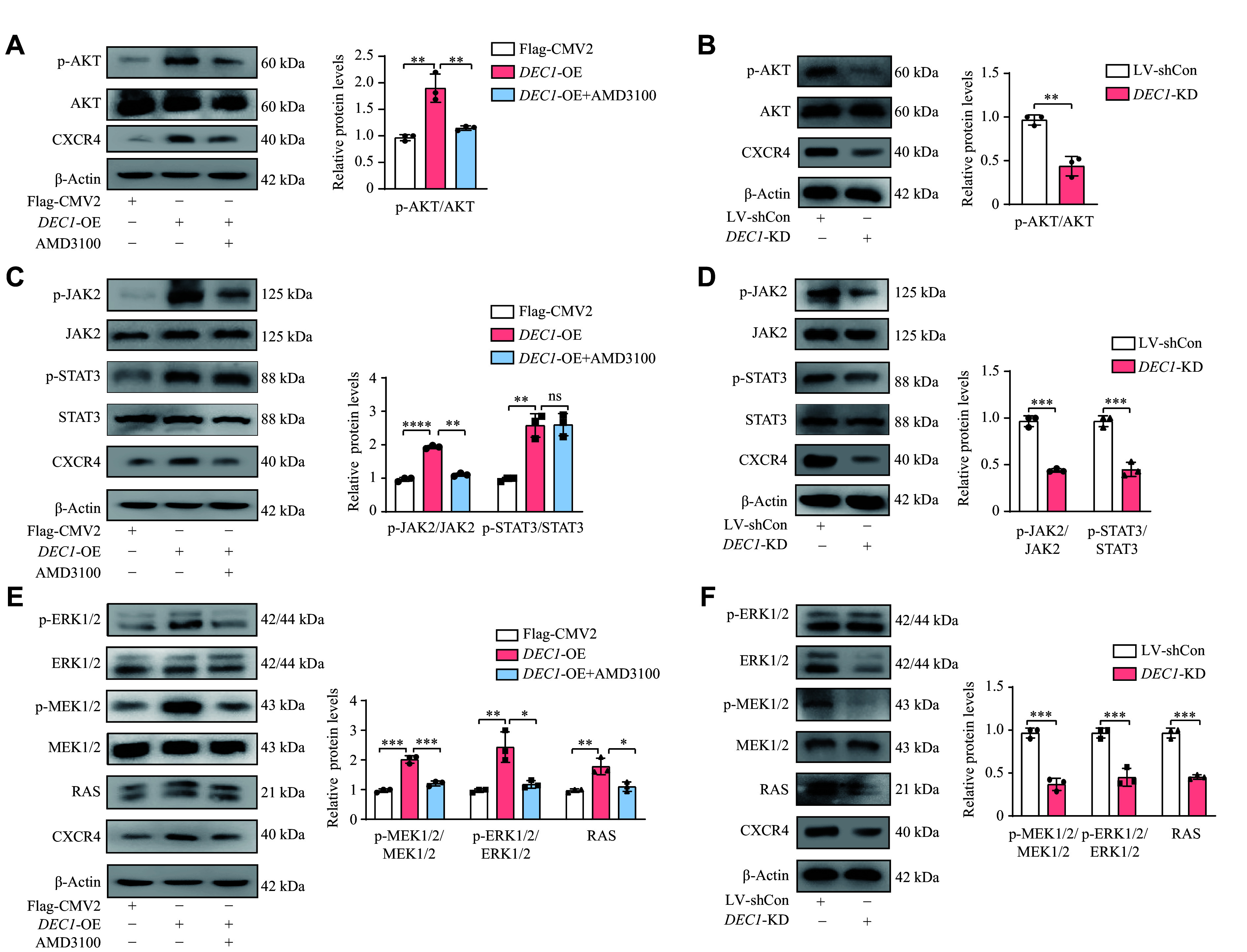
DEC1 upregulated CXCR4, thereby activating the p-AKT/AKT, JAK/STAT3, and p-ERK/ERK pathways in MDA-MB-231 cells. MDA-MB-231 cells were seeded into 6-well plates and cultured overnight. For *DEC1* overexpression (OE), the cells were transfected with either Flag-CMV2 or Flag- *DEC1*. The transfected cells were divided into three groups: Flag-CMV2, Flag- *DEC1*, and Flag- *DEC1* + AMD3100. Cells in the Flag- *DEC1* + AMD3100 group were treated with AMD3100, while the other two groups received 0.1% DMSO for 24 h. For *DEC1* knockdown (KD), cells were infected with LV-shCon or LV-sh *DEC1* for 24 h. Subsequently, cells from these five groups were harvested to analyze the expression of CXCR4 and p-AKT/AKT, JAK/STAT3, and p-ERK/ERK pathways by Western blotting. A–B: The impact of DEC1-OE and KD on CXCR4 expression and the p-AKT/AKT signaling pathway. C–D: The impact of DEC1-OE and KD on CXCR4 expression and the JAK/STAT3 signaling pathway. E–F: The impact of DEC1-OE and KD on CXCR4 expression and the p-ERK/ERK signaling pathway. Data are presented as mean ± standard deviation (all experiments were repeated at least three times). ^*^ *P* < 0.05, ^**^ *P* < 0.01, ^***^ *P* < 0.001, and ^ns^ *P* > 0.05, comparisons are shown in the figure. Data were analyzed using two-way ANOVA, and differences between groups were analyzed using Student's *t*-test.

*In vivo* signaling analysis revealed significantly reduced phosphorylation levels of AKT (p-AKT/AKT ratio), JAK2 (p-JAK2/JAK2), and ERK1/2 (p-ERK1/2/ERK1/2) in femoral metastases from *Dec1*-KD mice ( *Dec1*-KD 4T1) compared with *Dec1*-WT controls ( *Dec1*-WT 4T1) ( ***Supplementary Fig. 2***). These findings indicate that DEC1 promotes BC bone metastasis likely through the activation of PI3K/AKT, JAK2, and ERK signaling pathways. To further validate these observations, we performed parallel *in vitro* studies in BC cell lines. As shown in ***Fig. 7***, *DEC1*-OE significantly upregulated CXCR4 expression and enhanced phosphorylation of key signaling components (AKT, JAK2, ERK1/2, MEK1/2, and STAT3), while *DEC1*-KD produced the opposite effects. Notably, the CXCR4 antagonist AMD3100 significantly blocked DEC1-mediated activation of these pathways, demonstrating their CXCR4 dependence. Consistently, *DEC1*-OE reversed the *DEC1*-KD-mediated inhibition of PI3K/AKT, JAK/STAT, and MEK/ERK pathways activation ( ***Supplementary Fig. 4A***, available online) and the expression of their downstream proteins (vimentin, VEGF, MMP-9, and MMP-1) ( ***Supplementary Fig. 4B***, available online).

**Figure 8:**
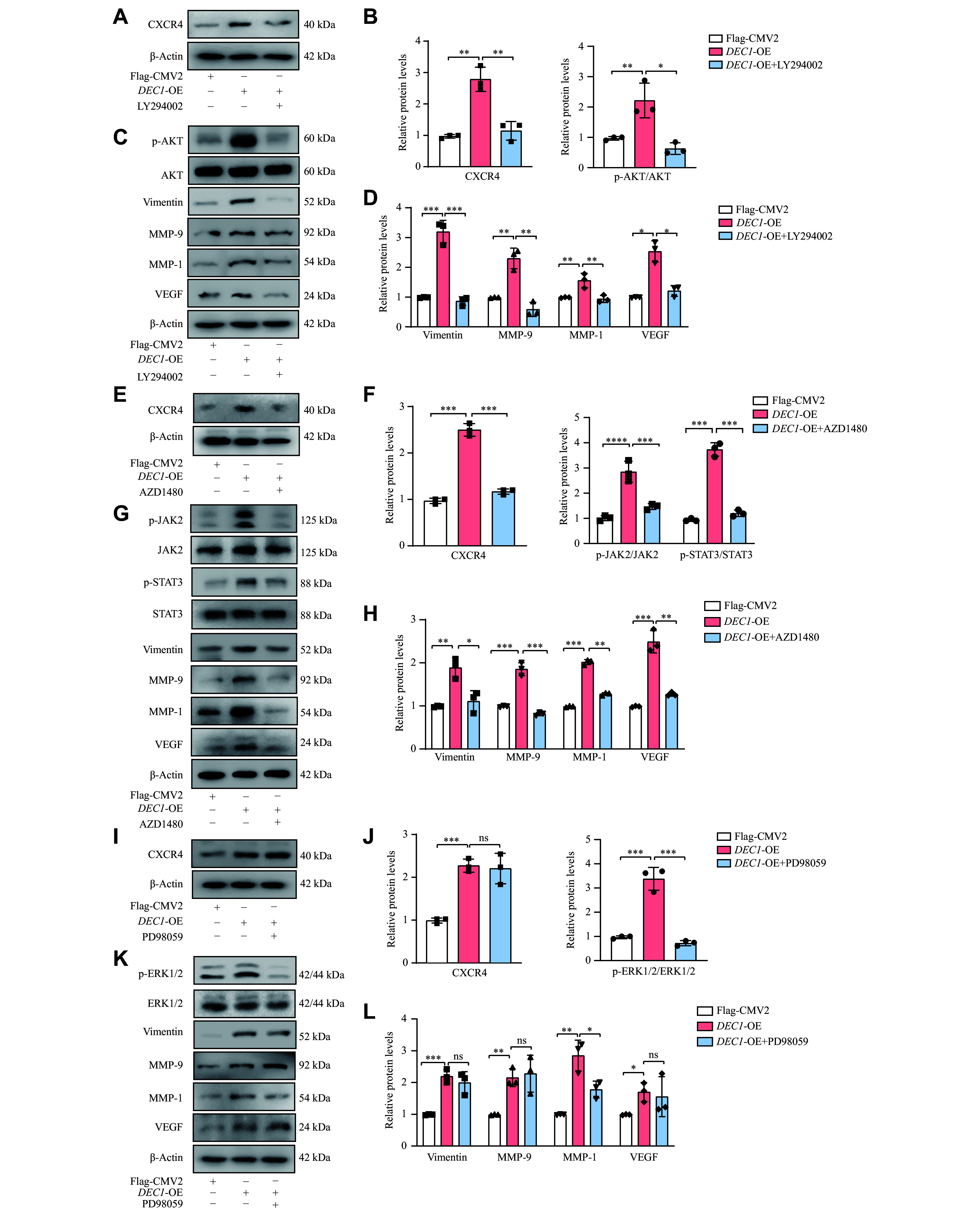
The p-AKT/AKT and JAK/STAT3 rather than p-ERK/ERK pathways were involved in the DEC1 upregulation of CXCR4 and the increases of vimentin, MMP1, MMP9, and VEGF in MDA-MB-231 cells. MDA-MB-231 cells were seeded into 6-well plates and cultured overnight. Cells were transfected with either Flag-CMV2 or Flag- *DEC1*. The transfected cells were divided into five groups: Flag-CMV2, Flag- *DEC1*, Flag- *DEC1* + LY294002, Flag- *DEC1* + AZD1480, and Flag- *DEC1* + PD98059. The first two groups were treated with an equal volume of 0.1% DMSO as a vehicle control, while the latter three groups were treated with LY294002 (2 μmol/L), AZD1480 (2 μmol/L), and PD98059 (5 μmol/L), respectively, for 24 h. Then, the cells from all groups were harvested to analyze the protein expression by Western blotting. A–D: Effects of LY294002 on the PI3K/AKT signaling pathway and on vimentin, VEGF, MMP-9, and MMP-1 expression induced by *DEC1* overexpression (OE). E–H: Effects of AZD1480 on the JAK/STAT3 signaling pathway and on vimentin, VEGF, MMP-9, and MMP-1 expression induced by *DEC*-OE. I–L: Effects of PD98059 on the p-ERK/ERK signaling pathway and on vimentin, VEGF, MMP-9, and MMP-1 expression induced by *DEC1*-OE. Data are presented as mean ± standard deviation (all experiments were repeated at least three times). ^*^ *P* < 0.05, ^**^ *P* < 0.01, ^***^ *P* < 0.001, and ^ns^ *P* > 0.05, comparisons are shown in the figure. Data were analyzed using two-way ANOVA, and differences between groups were analyzed using Student's *t*-test.

To systematically investigate the functional relationships between DEC1, CXCR4, and the signaling pathways, we employed a targeted pharmacological inhibition strategy using LY294002 (a PI3K inhibitor), AZD1480 (a JAK2 inhibitor), and PD98059 (a MEK/ERK inhibitor). As shown in ***Supplementary Fig. 5*** (available online), *DEC1*-OE significantly enhanced the migration of BC cells, while LY294002, AZD1480, and PD98059 attenuated this effect, particularly LY294002 and AZD1480. Further studies revealed that LY294002 and AZD1480 significantly abolished the increased p-AKT/AKT, p-JAK2/JAK2, and p-STAT3/STAT3 and their targets such as vimentin, VEGF, MMP-9, and MMP-1, as well as CXCR4 expression induced by *DEC1*-OE ( ***Fig. 8A***– ***8H***), but PD98059 did not do so ( ***Fig. 8I***– ***8L***). The findings indicate that DEC1 promotes the CXCR4 expression, consequently leading to increased levels of vimentin, VEGF, MMP-9, and MMP-1. These effects were primarily mediated by the PI3K/AKT and JAK2/STAT3 signaling pathways. Interestingly, inhibition experiments revealed that the PI3K/AKT and JAK2/STAT3 pathways, but not MEK/ERK, positively regulated CXCR4 expression, establishing a reinforcing signaling loop ( ***Fig. 8A***– ***8H***).

### DEC1 deficiency suppressed BC bone metastasis following intracardiac injection of 4T1 cells in mice, mediated through downregulation of the CXCR4/CXCL12 axis

**Figure 9:**
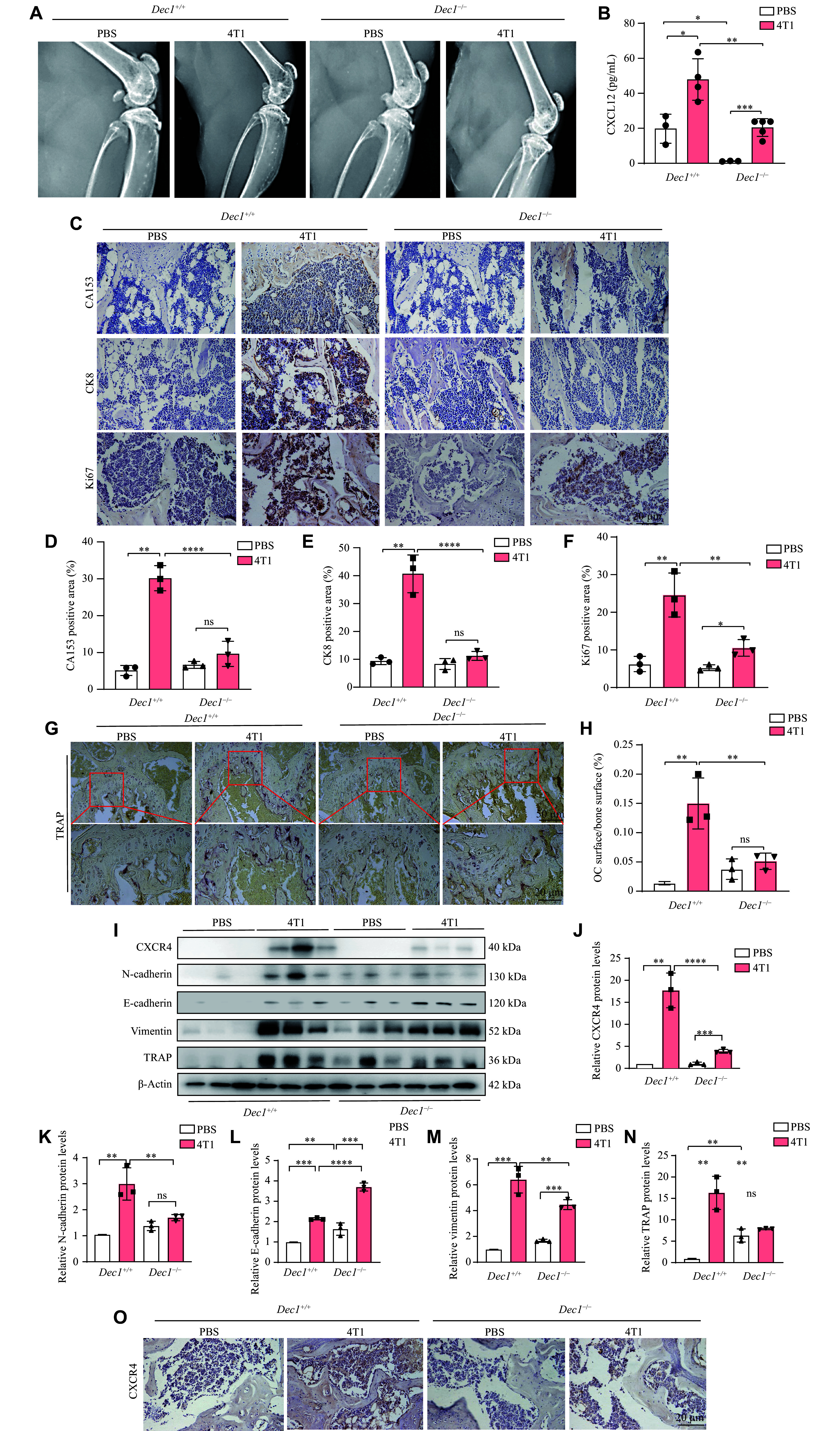
DEC1 deficiency prevented breast bone metastasis induced by intracardiac injections of 4T1 cells *via* decreasing CXCR4/CXCL12 in mice. Forty 4-month-old mice, comprising twenty *Dec1* ^+/+^ and twenty *Dec1* ^−/−^, were divided into four groups: *Dec1* ^+/+^-PBS, *Dec1* ^+/+^-4T1, *Dec1* ^−/−^-PBS, and *Dec1* ^−/−^-4T1. Mice in the *Dec1* ^+/+^-4T1 and *Dec1* ^−/−^-4T1 groups received an intracardiac injection of 4T1 mouse breast cancer cells to induce breast bone metastasis, while mice in the *Dec1* ^+/+^-PBS and *Dec1* ^−/−^-PBS groups received an equal volume of PBS *via* the same method for two months. *n* = 10 for each group. A: Osteolytic bone injury in the four groups of mice by X-ray. B: The serum CXCL12 amount in the four groups of mice ( *n* = 3–5). C–F: The CA153, CK8, and Ki67 expression in the four groups of mice by immunohistochemical staining ( *n* = 3). G and H: The TRAP-positive cells in the four groups of mice ( *n* = 3). I–N: The protein levels of CXCR4, TRAP, N-cadherin, E-cadherin, and vimentin in the four groups of mice by Western blotting ( *n* = 3). O: The CXCR4 expression in the four groups of mice. Data are presented as mean ± standard deviation. ^*^ *P* < 0.05, ^**^ *P* < 0.01, ^***^ *P* < 0.001, and ^ns^ *P* > 0.05, comparisons are shown in the figure. Data were analyzed using two-way ANOVA, and differences between groups were analyzed using Student's *t*-test.

To investigate how DEC1 modulates the tumor microenvironment to promote BC bone metastasis, we established a BC bone metastasis model by intracardiac injection of 4T1 cells into WT and *Dec1*-KO mice ( ***Supplementary Fig. 1D***), following a well-established protocol for studying BC metastasis to bone ^[ 34] ^. As shown in ***Fig. 9A***, *Dec1* ^+/+^-4T1 mice exhibited a significantly higher incidence of osteolytic lesions compared with *Dec1* ^−/−^-4T1 mice. Histological analysis further revealed that the femurs of *Dec1* ^+/+^-4T1 mice contained significantly greater numbers and larger areas of CA153-, CK8-, and Ki67-positive cells than those of *Dec1* ^−/−^-4T1 mice ( ***Fig. 9C***– ***9F***). Consistently, both the number and area of TRAP+ osteoclasts were significantly elevated in *Dec1* ^+/+^-4T1 mice than in *Dec1* ^−/−^-4T1 mice ( ***Fig. 9G*** and ***9H***), demonstrating the increased TRAP protein levels observed in *Dec1* ^+/+^-4T1 mice ( ***Fig. 9I*** [fifth line] and ***9N***). Furthermore, metastasis-associated proteins, including vimentin and N-cadherin, were significantly upregulated, whereas E-cadherin expression was significantly downregulated in *Dec1* ^+/+^-4T1 mice, compared with *Dec1* ^−/−^-4T1 mice ( ***Fig. 9I***– ***9N***). As anticipated, CXCR4 expression in the femur was also significantly higher in *Dec1* ^+/+^-4T1 mice than in *Dec1* ^−/−^-4T1 mice ( ***Fig. 9I*** [first line] and ***9O***). Additionally, while serum CXCL12 levels were elevated in both *Dec1* ^+/+^ and *Dec1* ^−/−^ tumor-bearing mice compared with their respective controls, *Dec1* ^+/+^-4T1 mice exhibited significantly higher CXCL12 levels than *Dec1* ^−/−^-4T1 mice ( ***Fig. 9B***). Notably, baseline CXCL12 levels in *Dec1* ^−/−^-PBS mice were significantly reduced compared with *Dec1* ^+/+^-PBS mice ( ***Fig. 9B***). Collectively, *Dec1* deficiency attenuated BC bone metastasis in mice following intracardiac injection of 4T1 cells, likely mediated by suppression of the CXCR4/CXCL12 signaling axis.

### DEC1 stimulated CXCL12 secretion from bone marrow stromal cells and osteoblasts

**Figure 10:**
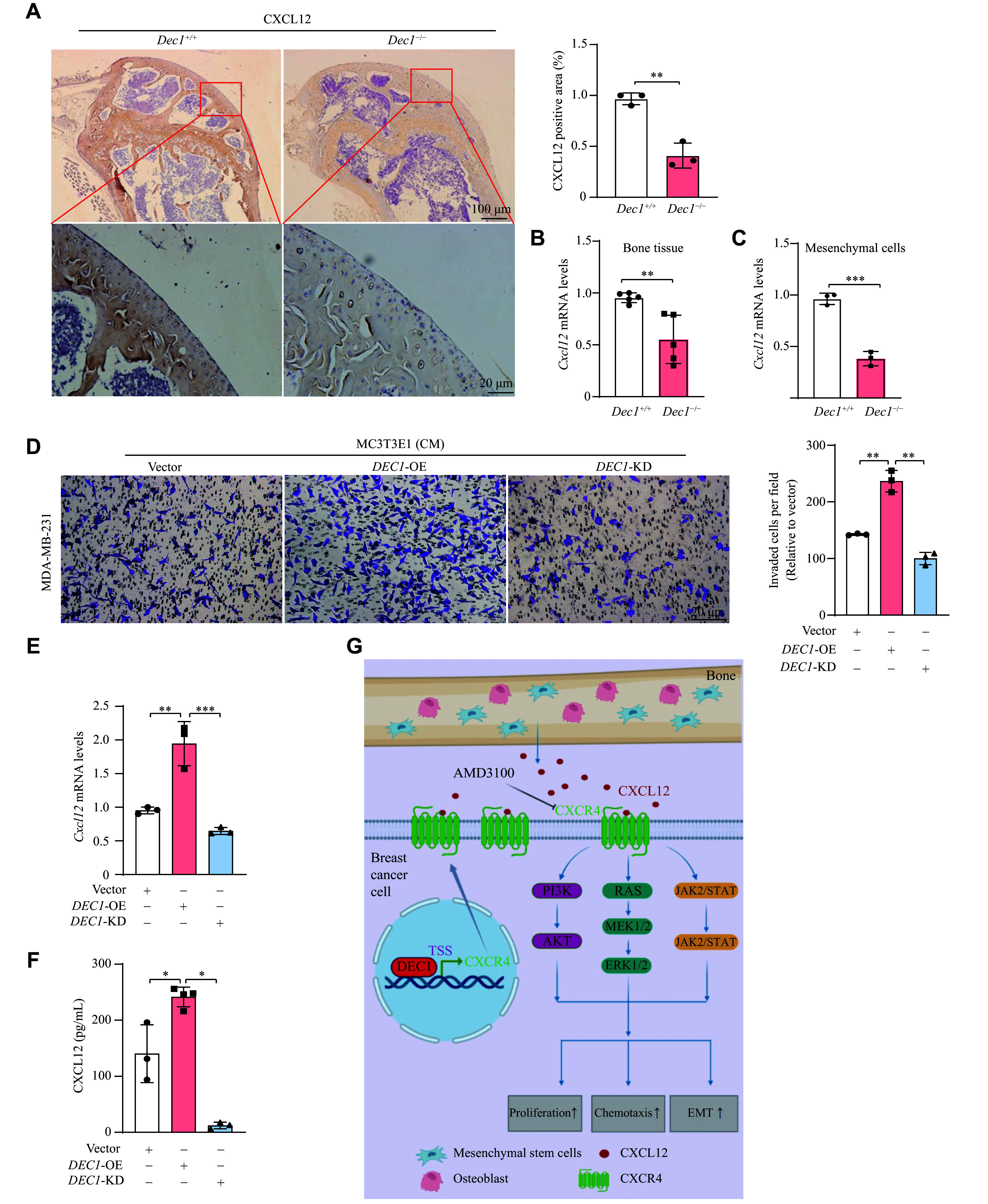
DEC1 promoted CXCL12 production from mesenchymal stromal cells in mice and MC3T3-E1 cells. A: The CXCL12 expression in the femur of wildtype (WT) and *Dec1*-knockout (KO) mice by immunohistochemical staining ( *n* = 3 in each group). B: The relative *Cxcl12* mRNA levels in bone tissues from the femur of WT and *Dec1*-KO mice ( *n* = 5 in each group). C: The relative *Cxcl12* mRNA levels in mesenchymal cells from the femur of WT and *Dec1*-KO mice ( *n* = 3 in each group). D–F: MC3T3-E1 cells were seeded into 6-well plates and cultured overnight. Cells were divided into three groups: Vector (infected with LV-shCon and transfected with Flag-CMV2), *DEC1*-OE (transfected with Flag- *DEC1*), and *DEC1*-KD (infected with LV-sh *DEC1*). After 24 h of transfection or infection, the culture medium from each group was collected, centrifuged, and filtered to obtain conditioned medium (CM). Effect of CM from MC3T3-E1 cells with *DEC1*-OE or KD on breast cancer cell migration by Transwell migration assays (D). The relative *Cxcl12* mRNA levels in MC3T3-E1 cells (E). The CXCL12 amount in CMs from *DEC1*-OE and KO in MC3T3-E1 cells by ELISA assays (F). G: Mechanism of DEC1 promoting breast cancer bone metastasis *via* transcriptional activation of CXCR4. Data are presented as mean ± standard deviation (all experiments were repeated at least three times). ^*^ *P* < 0.05, ^**^ *P* < 0.01, ^***^ *P* < 0.001, comparisons are shown in the figure. Data were analyzed using one-way ANOVA, and differences between groups were analyzed using Student's *t*-test. Abbreviation: EMT, epithelial-to-mesenchymal transition; KD, knockdown; OE, overexpression.

To elucidate the mechanism through which DEC1 in the bone microenvironment facilitates BC bone metastasis, we examined serum levels of CXCL12 (a CXCR4 ligand) in *Dec1* ^+/+^ and *Dec1* ^−/−^ mice. Consistent with the serum CXCL12 levels observed in the two mouse genotypes ( ***Fig. 9B***), IHC analysis revealed a significant reduction in CXCL12-positive cells in the femurs of *Dec1* ^−/−^-4T1 mice, compared with *Dec1* ^+/+^-4T1 mice ( ***Fig. 10A***). Similarly, *Cxcl12* mRNA levels in bone tissues and mesenchymal cells isolated from the femurs of *Dec1* ^−/−^-4T1 mice were significantly reduced compared with those from *Dec1* ^+/+^-4T1 mice ( ***Fig. 10B*** and ***10C***).

CXCL12 was primarily secreted by stromal cells and osteoblasts in the bone marrow, subsequently recruiting BC cells expressing high levels of CXCR4 to bone tissue ^[ 26] ^. Therefore, we employed conditioned medium from MC3T3-E1 cells (Vector control, *Dec1*-OE, and *Dec1*-KD) to investigate the role of DEC1 in modulating the bone microenvironment's influence on BC metastasis. As shown in ***Fig. 10D***, conditioned medium from *Dec1*-OE or *Dec1*-KD MC3T3-E1 cells enhanced or reduced the Transwell migration of MDA-MB-231 cells, respectively. Additionally, DEC1 manipulation increased or decreased *Cxcl12* mRNA expression in MC3T3-E1 cells, with parallel changes in CXCL12 protein levels in the conditioned medium ( ***Fig. 10E*** and ***10F***). These results indicate that DEC1 promotes BC metastasis by enhancing CXCL12 secretion from bone marrow stromal cells and osteoblasts.

## Discussion

Bone is the most common site for BC metastasis, occurring in 65%–80% of patients with BC bone metastasis ^[ 35] ^. The molecular mechanisms underlying bone metastasis, particularly the colonization and subsequent proliferation of BC cells in bone, are mediated by interactions between BC cells and the bone microenvironment ^[ 36– 37] ^. However, the precise mechanisms underlying BC bone metastasis remain to be elucidated. Certain transcription factors, such as RUNX2 ^[ 18] ^, NKX2-8 ^[ 19]^ and FOXF2 ^[ 38] ^, may contribute to this process. In the present study, using complementary approaches, we demonstrated that DEC1—a well-known transcription factor—enhanced BC cell proliferation, invasion, and transendothelial migration, thereby playing a critical role in BC bone metastasis in both clinical patient samples and an intracardiac BC cell injection model.

We provide compelling evidence of a close relationship between DEC1 and chemokine receptor CXCR4 in BC bone metastasis. (1) Database analysis, tissue microarrays, and patient samples revealed elevated DEC1 and CXCR4 expression in BC tissues compared with adjacent normal tissues. Notably, DEC1 expression was significantly higher in BC patients with distant metastasis than in those without metastasis. (2) Among tested cell lines (MDA-MB-231, MCF-7, and MCF-10A), both DEC1 and CXCR4 showed the highest expression in the highly metastatic MDA-MB-231 cells. (3) *DEC1* overexpression or knockdown correspondingly upregulated or downregulated CXCR4 expression in both human (MDA-MB-231, MCF-7, and SUM1315) and mouse (4T1) BC cell lines. (4) Intracardiac injection of *Dec1*-WT 4T1 cells (4T1- *Dec1*-WT) induced more severe osteolysis and higher DEC1/CXCR4 expression in femurs than *Dec1*-KD 4T1 cells (4T1- *Dec1*-KD). The 4T1- *Dec1*-WT group also showed increased levels of TRAP ^+^ osteoclasts and metastasis-related proteins (Ki67, MMP-9, MMP-1, N-cadherin, vimentin, SNAI1, and VEGF), alongside reduced E-cadherin ^[ 30, 39– 41] ^. (5) The metastasis-related protein changes observed *in vivo* were further validated *in vitro*. For example, *DEC1*-OE promoted BC cell proliferation and migration, accompanied by upregulation of CXCR4, N-cadherin, vimentin, and SNAI1, and downregulation of E-cadherin. These effects of *DEC1*-OE were abolished by AMD3100, a specific CXCR4 inhibitor. In contrast, *DEC1*-KD inhibited BC cell intravasation and migration, accompanied by decreased expression of CXCR4, N-cadherin, vimentin, and SNAI1, along with increased E-cadherin levels. Similarly, *DEC1*-OE upregulated the expression of proliferation- and stemness-related proteins, including Ki67, PCNA, NANOG, and OCT4, and these changes were also reversed by AMD3100 in MDA-MB-231 cells. Conversely, *DEC1*-KD reduced the levels of these proteins in MDA-MB-231 cells. Collectively, these findings indicate that DEC1 promotes BC bone metastasis by upregulating CXCR4, which in turn enhances proliferation, EMT, and stemness of breast cancer cells ^[ 39– 41] ^.

It is well established that the CXCR4/CXCL12 axis plays a pivotal role in BC bone metastasis ^[ 41– 42] ^. We demonstrated that CXCR4 expression was significantly reduced in *Dec1*-KD 4T1 cell intracardiac injection-induced BC bone metastasis mice compared with *Dec1*-WT 4T1 controls. Notably, both CXCR4 and DEC1 levels were significantly upregulated in highly metastatic triple-negative MDA-MB-231 cells, compared with MCF-7 cells ^[ 43] ^. *DEC1*-OE or *DEC1*-KD increased or decreased CXCR4 expression. Mechanistically, DEC1 did not affect *CXCR4* mRNA degradation but enhanced its transcription. ChIP-seq analysis revealed peak enrichment of *CXCR4* and *CXCL12*, along with DEC1 binding regions in the *CXCR4* promoter. Luciferase reporter assays ( *CXCR4*-Luc) further demonstrated that DEC1 bound to the *CXCR4* promoter within the −230 to −326 region, which contains two SP1 sites. Previous studies have shown that DEC1 represses transcription *via* E-box elements but activates it through SP1 sites ^[ 44– 45] ^. Additionally, the present study finds that the elevated DEC1 enhances CXCL12 secretion from bone marrow mesenchymal cells, the ligand for CXCR4. Collectively, these data indicate that DEC1 binds to the *CXCR4* promoter and induces its transactivation in BC cells, leading to the increased CXCR4 expression and enhanced BC cell survival in circulation ^[ 42] ^. Furthermore, *DEC1* promotes CXCL12 secretion from bone tissues, facilitating the homing of CXCR4-high BC cells to bone ^[ 12] ^.

CXCR4 activation has been shown to enhance proliferation, migration, and invasion *via* PI3K/AKT ^[ 46] ^, ERK ^[ 47] ^, and JAK/STAT3 signaling pathways. Our findings reveal a novel mechanism whereby DEC1 facilitates malignant progression (proliferation, migration, and invasion) and bone metastasis *via* CXCR4 upregulation, which is mechanistically linked to phosphorylation-dependent activation of the AKT, ERK1/2, and JAK2 signaling cascades. (1) Intracardiac injection of *Dec1*-WT 4T1 cells in the bone metastasis mouse model significantly upregulated phosphorylation levels of AKT, ERK1/2, and JAK2 in the femurs compared with PBS controls ( *P* < 0.05). These effects were attenuated in mice injected with *Dec1*-KD 4T1 cells. (2) Pharmacological inhibition of CXCR4 using AMD3100 significantly attenuated *DEC1*-OE-induced phosphorylation of AKT (p-AKT/AKT ratio decreased by 65%, *P* < 0.01), JAK2 (p-JAK2/JAK2 decreases by 72%, *P* < 0.01), and ERK1/2 (p-ERK1/2/ERK1/2 decreases by 68%, *P* < 0.01), demonstrating CXCR4's essential role in mediating these signaling events. (3) Pharmacological inhibition experiments revealed distinct pathway dependencies: LY294002 suppressed DEC1-induced CXCR4 upregulation ( *P* < 0.01), PI3K/AKT (p-AKT/AKT, *P* < 0.05), and metastasis-associated proteins such as vimentin, MMP-9, MMP-1, and VEGF ( *P* < 0.0001, 0.01, 0.05); AZD1480 similarly attenuated CXCR4 expression ( *P* < 0.001), JAK2 (p-JAK2/JAK2, *P* < 0.001), and downstream effectors; whereas PD98059 showed no significant effects on these parameters (all *P* > 0.05). These results demonstrate a positive feedback mechanism whereby DEC1 enhances CXCR4 expression through PI3K/AKT- and JAK2-mediated signaling pathways, independent of ERK activation. The mechanism by which PI3K/AKT and JAK2 inhibitors attenuated DEC1-induced CXCR4 expression remains unclear and warrants further investigation in future research. Consistent with previous reports, our findings align with established mechanisms whereby CXCR4 promotes malignant phenotypes (proliferation, migration, and invasion) through activation of key signaling pathways including PI3K/AKT (p-AKT/AKT), JAK/STAT (p-JAK2/JAK2), and MAPK/ERK (p-ERK1/2/ERK1/2) ^[ 48– 51] ^. Notably, these pathways reciprocally regulate CXCR4 expression, forming a positive feedback loop that amplifies oncogenic signaling. The mechanisms underlying this reciprocal regulation of CXCR4 expression will be investigated in our future study. Collectively, our findings demonstrate that DEC1 promotes BC bone metastasis by directly transactivating CXCR4 expression and potentiating the CXCR4/CXCL12 axis through PI3K/AKT- and JAK2-mediated positive feedback mechanisms. The findings provide a molecular basis for targeting DEC1 to prevent and treat BC bone metastasis ( ***Fig. 10G***).

This study has an inherent limitation regarding the animal model: While the *Dec1-*knockout mice were on a C57BL/6 background, we established the BC bone metastasis model through intracardiac injection of 4T1 cells (derived from BALB/c mice) at a relatively high dose (1 × 10 ^6^) with an extended observation period (60 days) in C57BL/6 mice. Although this cross-strain modeling approach has been adopted in some previous studies ^[ 52] ^, potential allogeneic immune responses cannot be completely ruled out. Nevertheless, the remarkable consistency between our *in vivo* results and both clinical observations and cellular experiments strongly supports the biological relevance of our findings.

In conclusion, our findings demonstrate that DEC1 promotes BC bone metastasis by directly transactivating CXCR4 expression and potentiating the CXCR4/CXCL12 axis through PI3K/AKT- and JAK2-mediated positive feedback mechanisms.

## SUPPLEMENTARY DATA

Supplementary data to this article can be found online.
