# Supplementary material for: DEC1 promotes breast cancer bone metastasis through transcriptional activation of CXCR4
Source: J Biomed Res. 2025 May 27;39(5):478–99. doi: 10.7555/JBR.39.20250031 (PMC12481698; doi:10.7555/JBR.39.20250031)
Supplement: Supplementary file 1 — Supplementary data to this article can be found online. [file jbr-39-5-478-Supplementary.pdf]

Available online at www.jbr-pub.org.cn

Open Access at PubMed Central

Journal of Biomedical Research, 2025 39(5): 1–4

JBR

Supplementary Data

DEC1 promotes breast cancer bone metastasis through transcriptional activation of CXCR4

Ying Huo, Kaiao Chen, Zhiyi Qiang, Lan Lin, Wei Liu✉, Jian Yang✉

Department of Pharmacology, Nanjing Medical University, Nanjing, Jiangsu 210066, China.

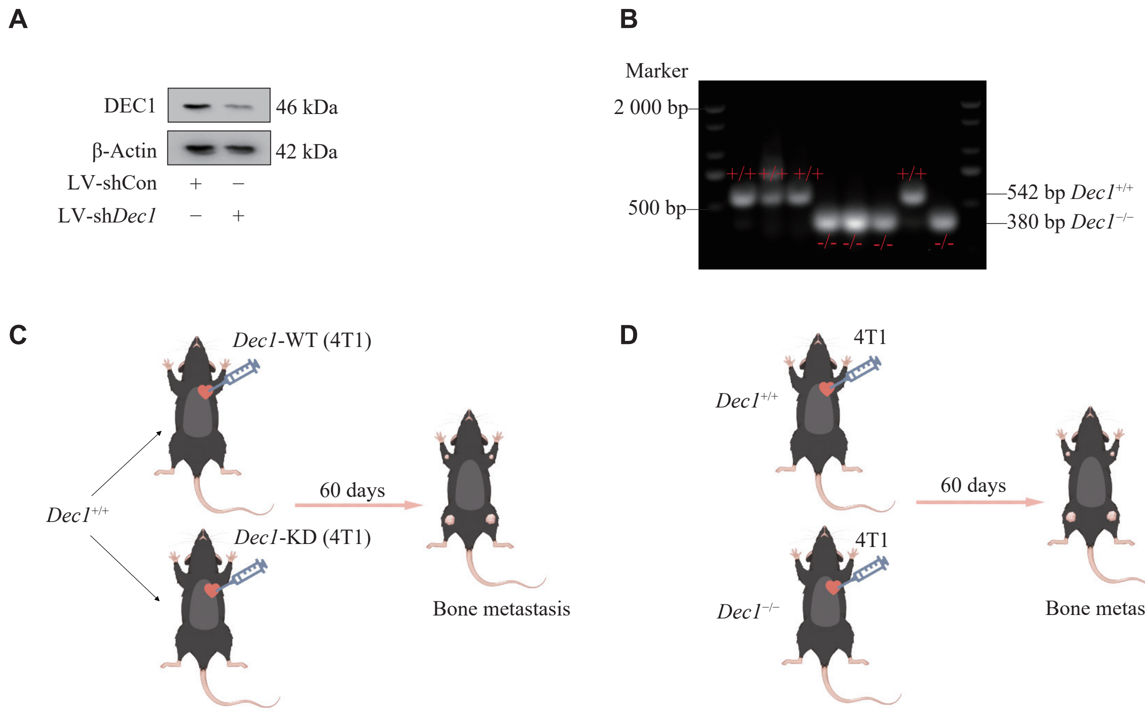

Supplementary Fig. 1 **The flow chart of making a BC bone metastasis model with intracardiac injection in mice and genotype identification of mice.** A: The knockdown of DEC1 in 4T1-*Dec1*- cells (4T1-*Dec1*-WT [LV-shCon] or 4T1-*Dec1*-KD [LV-sh*Dec1*]) was validated by Western blotting. B: Genotype identification of mouse tail DNA derived from *Dec1*$^{+/+}$ and *Dec1*$^{-/-}$ mice was extracted and analyzed by using PCR for genotype identification. The protocol, primers, and reagents were provided by RIKEN BioResource Center. *Dec1*$^{+/+}$ and *Dec1*$^{-/-}$ mice are shown in the figure. C and D: The flow chart of making a BC bone metastasis model with intracardiac injection in mice.

✉Corresponding authors: Wei Liu and Jiang Yang, Department of Pharmacology, Nanjing Medical University, 101 Longmian Avenue, Nanjing, Jiangsu 210066, China. E-mails: liuweicp@njmu.edu.cn (Liu) and jianyang@njmu.edu.cn (Yang).

Received: 24 February 2025; Revised: 06 May 2025; Accepted: 08 May 2025; Published online: 27 May 2025

CLC number: R737.9, Document code: A

The authors reported no conflict of interests.

This is an open access article under the Creative Commons Attribution (CC BY 4.0) license, which permits others to distribute, remix, adapt and build upon this work, for commercial use, provided the original work is properly cited.

© 2025 by Journal of Biomedical Research.

https://doi.org/10.7555/JBR.39.20250031

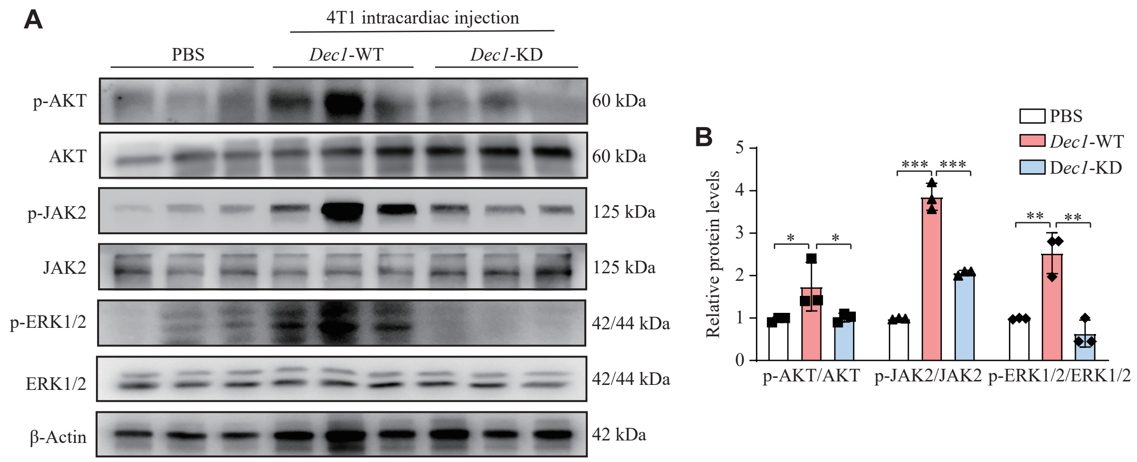

Supplementary Fig. 2 **Knockdown of *Dec1* in 4T1 cells decreased p-AKT/AKT, p-JAK2/JAK2, and p-ERK/ERK levels in mice.** A and B: We made BC bone metastasis models with the intracardiac injection of PBS, 4T1-WT, or 4T1-KD in mice. Two months later, the proteins were extracted from bone tissue in the three groups. Then, p-AKT/AKT, p-JAK2/JAK2, and p-ERK/ERK were detected by Western blot. Data are presented as mean ± standard deviation (n = 3 in each group). $^{*}P < 0.05$, $^{**}P < 0.01$, $^{***}P < 0.001$, comparisons are shown in the figure. Data were analyzed using two-way ANOVA, and differences between groups were analyzed using Student's t-test.

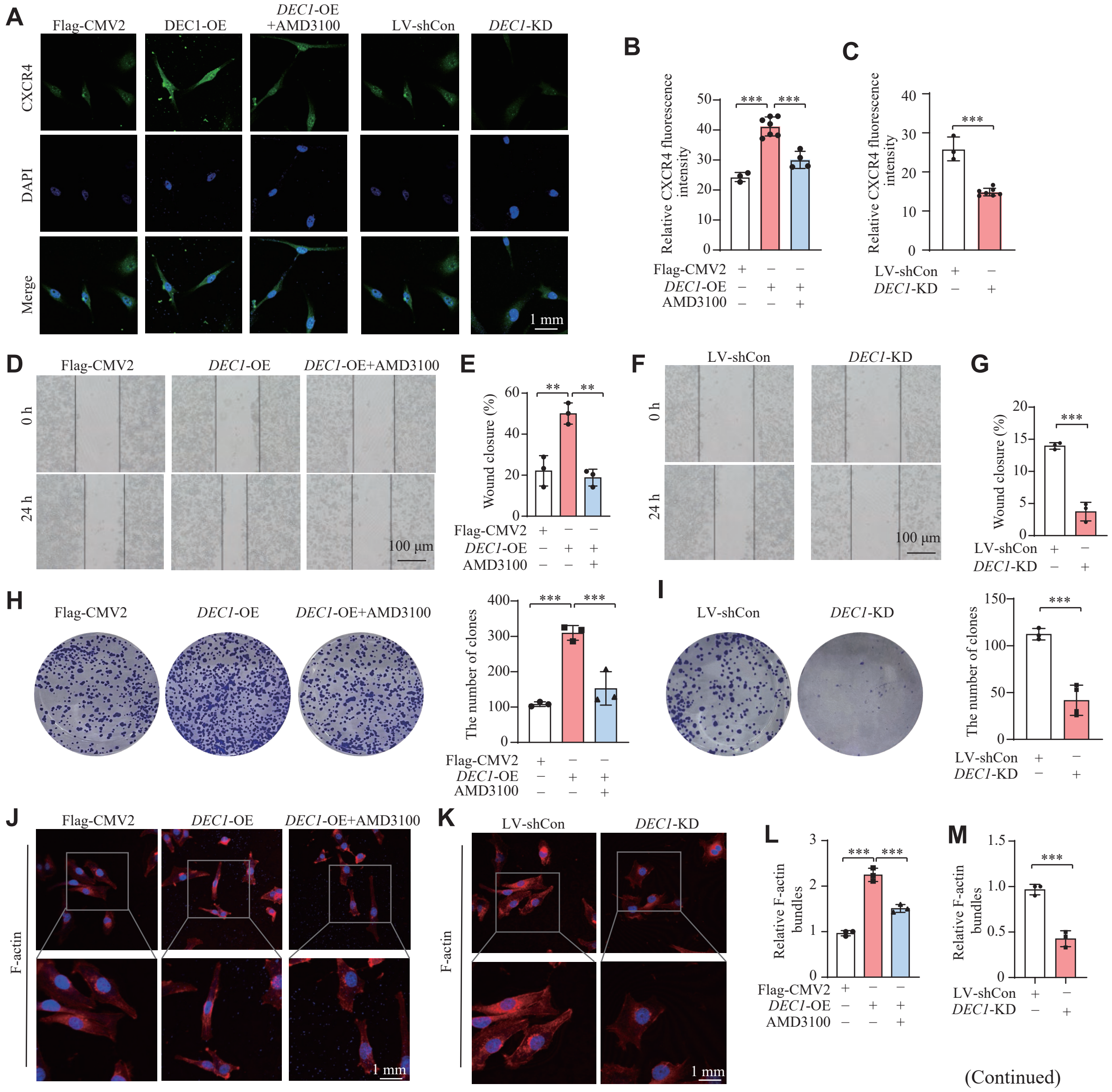

(Continued)

(Continued)

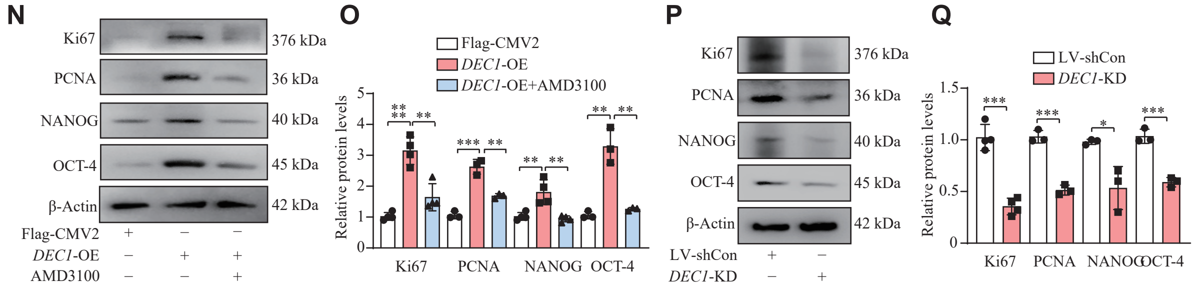

Supplementary Fig. 3 **CXCR4 was involved in the promoted proliferation by DEC1 in MDA-MB-231 cells.** MDA-MB-231 cells were seeded into 6-well plates and cultured overnight. For DEC1 overexpression (OE), the cells were transfected with either Flag-CMV2 or Flag-*DEC1* plasmids. The transfected cells were then divided into three groups: Flag-CMV2, Flag-*DEC1*, and Flag-*DEC1* + AMD3100. Cells in the Flag-*DEC1* + AMD3100 group were treated with AMD3100, whereas the other two groups received DMSO for 24 h. For *DEC1* knockdown (KD), cells were infected with LV-shCon or LV-sh*DEC1* for 24 h. A–C: The impact of *DEC1*-OE and KD on CXCR4 expression by immunofluorescence staining. Scale bar = 1 mm (laser confocal). D–G: The impact of *DEC1*-OE and KD on cell wound healing. Scale bar = 100 μm. H–I: The impact of *DEC1*-OE and KD on the colony formation. J–M: The impact of *DEC1*-OE and KD on the F-actin formation. Scale bar = 1 mm (laser confocal). N–Q: The impact of *DEC1*-OE and KD on the expression of Ki67, PCNA, NANOG, and OCT-4 by Western blotting. Data are presented as mean ± standard deviation (all experiments were repeated at least three times). $^{*}P < 0.05$, $^{**}P < 0.01$, $^{***}P < 0.001$, comparisons are shown in the figure. Data were analyzed using two-way ANOVA, and differences between groups were analyzed using Student's *t*-test.

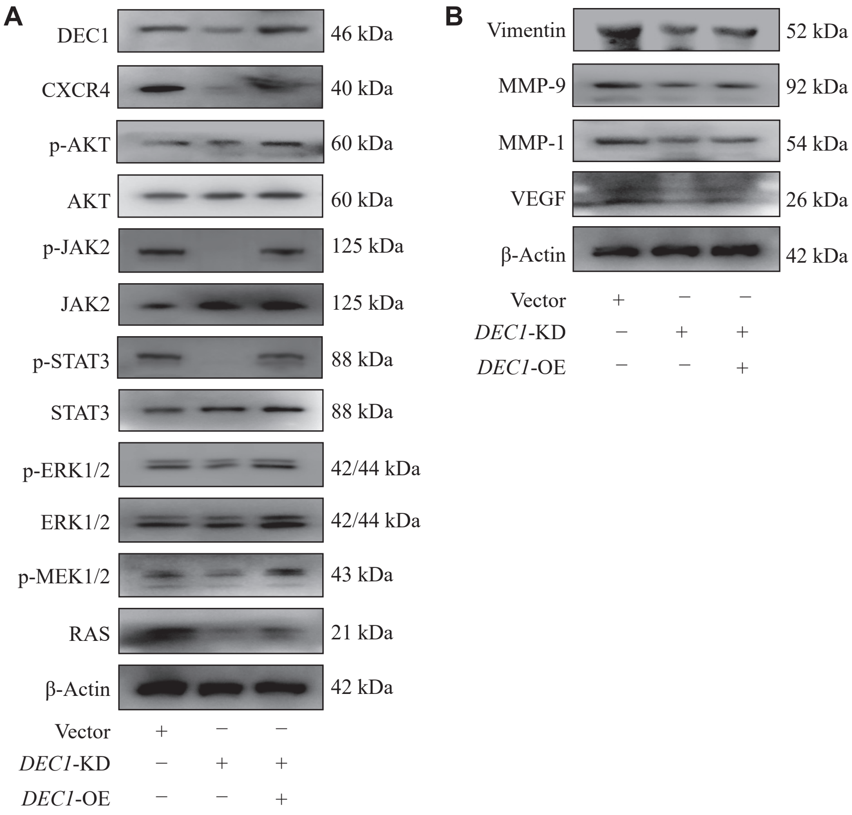

Supplementary Fig. 4 **Forced expression of DEC1 successfully reversed both the signaling pathway alterations and downstream gene expression changes caused by *DEC1*-KD in MDA-MB-231 cells.** A–B: MDA-MB-231 cells were seeded into 6-well plates and cultured overnight. Cells were divided into three groups: Vector (infected with LV-shCon and transfected with Flag-CMV2), *DEC1*-KD (infected with LV-sh*DEC1*), and *DEC1*-KD + *DEC1*-OE (infected with LV-sh*DEC1* + transfected with Flag-*DEC1*) for 24 h. Subsequently, cells from these groups were harvested to analyze the CXCR4 expression, p-AKT/AKT, p-JAK2/JAK2, p-ERK/ERK, and EMT markers (vimentin, MMP9, MMP1, VEGF) by Western blotting. $^{*}P < 0.05$, $^{**}P < 0.01$, $^{***}P < 0.001$, comparisons are shown in the figure. Data were analyzed using two-way ANOVA, and differences between groups were analyzed using Student's *t*-test.

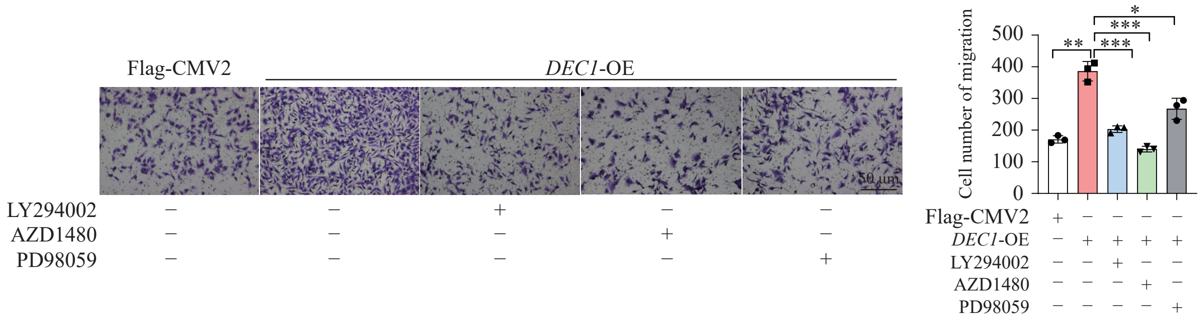

Supplementary Fig. 5 **The effects of LY294002 (a PI3K inhibitor), AZD1480 (a JAK2 inhibitor), and PD98059 (a MEK/ERK inhibitor) on enhancing the migration of MDA-MB-231 cells induced by DEC1-OE.** MDA-MB-231 cells were seeded into 6-well plates and cultured overnight. Cells were transfected with either Flag-CMV2 or Flag-*DEC1*. The transfected cells were divided into five groups: Flag-CMV2, Flag-*DEC1*, Flag-*DEC1* + LY294002, Flag-*DEC1* + AZD1480, and Flag-*DEC1* + PD98059. The first two groups were treated with an equal volume of 0.1% DMSO as a vehicle control, while the latter three groups were treated with LY294002 (2 μmol/L), AZD1480 (1 μmol/L), and PD98059 (5 μmol/L), respectively. Cell migration assays were performed. $^{*}P < 0.05$, $^{**}P < 0.01$, $^{***}P < 0.001$, comparisons are shown in the figure. Data were analyzed using two-way ANOVA, and differences between groups were analyzed using Student's *t*-test.
